# A European Association for Palliative Care White Paper defining an integrative palliative, geriatric, and rehabilitative approach to care and support for older people living with frailty and their family carers: a 28-country Delphi study and recommendations

**DOI:** 10.1016/j.eclinm.2025.103403

**Published:** 2025-08-12

**Authors:** Lieve Van den Block, Kim de Nooijer, Sophie Pautex, Lara Pivodic, Nele Van Den Noortgate, Caroline Nicholson, Katarzyna Szczerbińska, Sandra Martins Pereira, Rebecca Tiberini, Barbara Hanratty, Rose Miranda

**Affiliations:** a End-of-Life Care Research Group, Vrije Universiteit Brussel (VUB) & Universiteit Gent, Brussels, Belgium; b Department of Family Medicine and Chronic Care, Vrije Universiteit Brussel (VUB), Brussels, Belgium; c Division of Palliative Medicine, Department of Rehabilitation and Geriatrics, University Hospital Geneva and University of Geneva, Switzerland; d Department of Geriatric Medicine and Palliative Care, Ghent University Hospital, Ghent, Belgium; e Faculty of Health and Medical Sciences, University of Surrey, Guildford, United Kingdom; f Laboratory for Research on Ageing Society, Chair of Epidemiology and Preventive Medicine, Medical Faculty, Jagiellonian University Medical College, Krakow, Poland; g Department of Internal Medicine and Geriatrics, University Hospital in Krakow, Poland; h Universidade dos Açores | Fundação Gaspar Frutuoso, CEEAplA: Center of Applied Economic Studies of the Atlantic, Ponta Delgada, São Miguel Island, Azores, Portugal; i Independent Consultant in Rehabilitative Palliative Care and Strategic Leadership, St. Moritz, Switzerland; j Population Health Sciences Institute, Faculty of Medical Sciences, Newcastle University, Newcastle, United Kingdom

**Keywords:** Frailty, Aging, Palliative care, Geriatrics, Rehabilitation

## Abstract

**Background:**

A fast-growing number of older people living with frailty experience complex needs throughout their illness trajectory. However, currently, there is no international consensus on optimal care and support to older people living with frailty and their family carers. The European Association for Palliative Care (EAPC) Reference Group on Aging and Palliative Care aimed to develop a White Paper defining an optimal integrative palliative, geriatric, and rehabilitative approach to care and support for this population.

**Methods:**

We conducted an international Delphi study, comprising an iterative preparatory phase using literature and input from international and interdisciplinary experts in research, practice, and policy, (between 2020 and 2022) and an online consensus-based survey (2023) with 63 professional experts and 19 older person's representatives from 28 countries in Asia, Australia, Europe, and North America.

**Findings:**

The EAPC White Paper comprises 11 key domains that capitalize on the strengths of palliative care, geriatrics, and rehabilitation; and 34 key recommendations that elucidate what is needed from clinical, health service, and public health perspectives to address the multidimensional needs of this population, support their capacities, and maintain their quality of life and well-being until the end of life, including bereavement of carers.

**Interpretation:**

This EAPC White Paper presents a gold standard for the care and support for older people living with frailty and their family carers. It calls for a radical shift in healthcare provision to effectively integrate palliative, geriatric, and rehabilitative approaches to care and support for this population and represents a first critical step in establishing how to achieve this.

**Funding:**

10.13039/501100003130Research Foundation Flanders Belgium funded the postdoctoral mandates of RM (12D4523N) and KDN (12AEO24N).

**Research in context**

**Evidence before this study**

Before undertaking the Delphi survey, we conducted literature reviews using database searches in PubMed and Google Scholar (from inception up to January 2022) with keywords related to older people living with frailty (“frailty”, “elderly”, “older people”, “older persons”, “older adults” OR “older population”), in combination with keywords for palliative care (“palliative care”, “end-of-life care”, “terminal care”, “death”, OR “dying”). Additionally, we used citation tracking in Google Scholar; conducted a search in an Australian database (caresearch.com.au); and used suggestions from the core author group. We found no publications that integrated the existing evidence in aging and frailty across the disciplines of geriatrics, rehabilitation and palliative care; nor provided an evidence- and consensus-based framework that defines an integrative palliative, geriatric, and rehabilitative approach to care and support for older people living with frailty and their family carers.

**Added value of this study**

Full expert consensus was reached on 34 recommendations captured under 11 domains. This White Paper elucidates what is needed from clinical, health service, and public health perspectives to address the multidimensional needs of this population, support their capacities, and maintain their quality of life and well-being until the end of life, including bereavement of their family carers.

**Implications of all the available evidence**

The integrative palliative, geriatric, and rehabilitative approach to care and support comprising this EAPC White Paper presents a gold standard for the care and support for older people living with frailty and their family carers. This White Paper calls for a radical shift in healthcare provision to effectively integrate palliative, geriatric and rehabilitative approaches to care and support for this population and represents a first critical step in establishing how to achieve this. Finally, this work should drive future quality improvement efforts, research, and policy, aiming to improve what older people living with frailty and their family carers value.

## Introduction

A fast-growing number of older people live with frailty.^1^ Frailty, according to medical literature, is a clinical syndrome resulting from altered metabolism coupled with abnormal stress responses,^2^, ^3^, ^4^ or alternatively as a staged deficit accumulation focusing on a state of poor health due to compounded age-related deficits.^3^, ^4^, ^5^ Frailty is characterized by a decline in function across multiple organ systems and associated with a wide range of adverse health outcomes, including hospitalization, falls and fractures, multimorbidity, disability, and mortality.^6^ It has been associated with cognitive decline, complicating communication and decision-making processes,^3^^,^^4^^,^^7^ and older people living with frailty may experience several social, psychological or existential needs throughout their trajectory.^8^, ^9^, ^10^ They may require intensive and prolonged care and support, and often strongly rely on their family carers, who might in turn experience heightened distress and burden from their caregiving role.^10^^,^^11^

Over the past decades, several disciplines have explored the provision of optimal care and support for older people living with frailty and their family carers, each through the unique lens of their specialism.^12^ As part of the work of the European Association for Palliative Care (EAPC) Reference Group on Aging and Palliative Care, we aimed to develop an international White Paper that spans across disciplines by defining an integrative palliative, geriatric, and rehabilitative approach to care and support for this growing population of patients. The selection of these three disciplines is grounded primarily in the population itself. Caring for older people living with frailty is a main focus of each of these disciplines; albeit each in a different way, and typically at different stages of the illness trajectory.^13^ Whilst palliative care, geriatric, and rehabilitative disciplines represent distinct specialisms, they share key principles and approaches. Compared to geriatrics or rehabilitation, the role of general or specialist approaches to palliative care in frailty has been least developed.^12^^,^^14^ Nonetheless, clear clinical links between some of the disciplines have been established,^15^^,^^16^ and a framework guiding future efforts for all three disciplines would signify clear progress towards further integration. To date, the evidence from the past decades of research on frailty care and support has not been integrated across these disciplines in an evidence- and consensus-based integrative framework.^12^ Within our international White Paper on Frailty, we bring this knowledge together, overcoming the specialty silos between palliative care, geriatrics, and rehabilitation, capitalizing on the strengths of each discipline.

This article reports the development and outcomes of a 28-country Delphi study aiming to develop a White Paper defining an integrative palliative, geriatric, and rehabilitative approach to care and support on frailty; and involving a wide range of professional experts, disciplines, and representatives of older persons. This work aims to produce recommendations that could drive quality improvement efforts, further research, and policy to improve the lives of older people living with frailty and their family carers.

## Methods

### Design

**Fig. 1:**
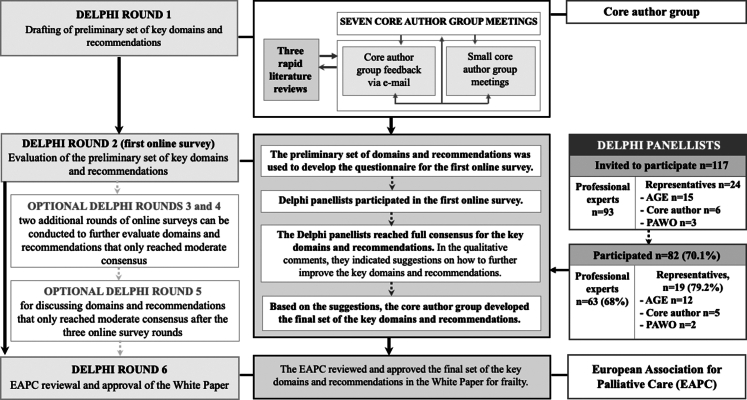
**Flowchart of the Delphi study**.

We conducted a Delphi study, an established formal consensus method that considers empirical evidence and gathers collective opinions of experts through a systematic, multiple round process where each stage is built on the results of the previous one (Fig. 1).^17^ This study was conducted by the core members of the EAPC Reference Group on Aging and Palliative Care, described hereafter as the core author group. It adhered to the Guidance on Conducting and Reporting Delphi Studies in palliative care (CREDES).^18^

Delphi round 1 (see Fig. 1 and Appendix 1 for details) was an iterative preparatory process using three cycles of rapid literature reviews until January 2022 and seven core author group discussions between January 2020 and April 2022. The literature reviews provided relevant empirical evidence, including evidence from the perspectives and needs of older persons themselves and those living with frailty and their family carers; although the evidence base for people living with severe frailty seemed limited.^19^ The core author group discussed and scrutinized the empirical evidence. LVDB and RM conducted meetings at multiple strategic timepoints to consolidate the evidence with the core author input. This round resulted in the preliminary list of key domains and recommendations, which was the basis for the questionnaire that we developed and piloted for the online survey in Delphi round 2.

Delphi round 2 was the first online survey with international Delphi panellists, conducted between April and June 2023. The survey questionnaire included questions on age, gender, highest educational attainment, participants' self-rated experience, professional background, and a quantitative evaluation of the preliminary list of domains and recommendations for practice, research, and policy (see Appendix 2). It allowed for qualitative comments on how to improve the domains and recommendations. To identify new empirical evidence and to complement the earlier cycles of the rapid reviews, an update of the rapid literature review was conducted until February 2025. Delphi rounds 3–5 were foreseen as optional in case consensus was not reached. In Delphi round 6, the EAPC Board of Directors provided feedback and approved this White Paper (23 April 2025).

### Participants and recruitment

The core author group represented multiple disciplines in primary, secondary and tertiary care, including experts on frailty, palliative care, geriatrics, and/or rehabilitation, based on research, education and/or their clinical experience, from six countries, with expertise spanning multiple care settings (home, nursing home, and hospital). We aimed to include a diverse and relevant panel that reflects the breadth of perspectives, knowledge, and disciplines needed for this research topic. The Delphi panellists were professional experts and representatives of older persons and their family carers, who in this study are either older persons themselves, family carer of older persons, or represent the perspectives of older persons. Table 1 explains the participants' eligibility criteria and recruitment. We recruited professional experts through the EAPC Aging and Palliative Care Reference Group's network members (n = 111), and representatives of older people and their family carers through different channels:

- AGE Platform Europe (an EU network of non-profit organizations of and for older people) distributed the survey to their taskforce members on dignified and healthy aging, comprising older persons and/or carers of older person from different European member organizations.
- The Health Innovation and Research Institute's Patient Advisory Council of Ghent University Hospital in Belgium was asked to distribute to older persons' representatives.
- The core author group contacted older persons themselves, i.e., who were part of PPI groups in their ongoing research.

**Table 1:** The eligibility criteria and recruitment of the Delphi panellists.

Eligibility criteria	Recruitment details
**Professional experts** • aged 18 years and older • expertise in palliative care, geriatrics, and/or rehabilitation for older people and those living with frailty through practice, policy, research or clinical experience • a healthcare professional/clinical practitioner, policymaker and/or researcher • capable of understanding, reading, and writing English • capable to provide informed consent to participate in the online survey • agreed to the use of their data for research purposes, indicated by checking a box in the first page of the online survey. Those who did not check this box could not proceed to the online survey questionnaire.	We recruited the professional experts via the EAPC Reference Group on Aging and Palliative Care Network (n = 111). We started building this network in 2021, first by identifying and inviting candidate members from our networks who are experts in palliative care, geriatrics, and rehabilitation. Second, we checked the reference lists of relevant meta-reviews and reviews to invite other experts outside of our professional network, and to achieve balance in expert profession and country of residence. The Reference Group network members are researchers, clinical practitioners, and/or policymakers from countries in Asia, Australia, Europe, North America, and Zealandia. We focused on high-income countries, as their resources and priorities of healthcare strategies and policies in terms of aging and frailty are most likely different from low- or middle-income countries. To become a network member, the candidate member completed an online membership form, wherein they could indicate if they agree to participate in this Delphi study (yes/no/I would like more information). A specially designed database containing the current information about the Reference Group Network was created in MS Excel version 16 (©Microsoft 2022) and stored in the fully secured professional Microsoft SharePoint of the Vrije Universiteit Brussel. To secure the privacy and confidentiality of the network members, this database was only accessible to the researcher (RM) responsible for the e-mail correspondences with the network members and an assistant (CV) who was responsible for assisting the data collection for the online surveys.
**Older person's representatives** • represent older persons and/or their family carers • aged 18 years and older • is capable of understanding, reading and writing English • is capable to provide informed consent to participate in the online survey • agreed to the use of their data for research purposes, indicated by checking a box in the first page of the online survey. Those who did not check this box could not proceed to the online survey questionnaire.	The older person's representatives either older persons themselves, family carer of older persons, or represent the perspectives of older persons. They were recruited through different channels: - AGE Platform Europe (an EU network of non-profit organizations of and for older people) distributed the survey to their taskforce members on dignified and healthy aging, comprising older persons and/or carers of older person from different European member organizations - the Health Innovation and Research Institute's Patient Advisory Council of Ghent University Hospital in Belgium was asked to distribute to older persons' representatives - the core author group contacted older persons themselves, i.e., who were part of PPI groups in their ongoing research. The AGE coordinator, the coordinator of the Patient's Advisory Council of Ghent University, and the core authors sent an invitation e-mail with information about the study to the identified eligible representatives.

### Endpoint and scores

The main endpoint was consensus on key domains and recommendations for an integrative palliative, geriatric, and rehabilitative approach to care and support for older people living with frailty and their family carers. The processing of scores to determine the extent of consensus (Appendix 3) was based on the scoring used in analogous studies.^20^^,^^21^ The Delphi panellists evaluated the importance of the preliminary set of key domains and each individual domain using a 10-point importance scale, where 1 was ‘not important’ and 10 was ‘very important’. The recommendations were positively formulated in the questionnaire and evaluated on a 5-point agreement scale: strongly disagree (1); moderately disagree (2); neither agree nor disagree (3); moderately agree (4); and strongly agree (5).

### Data collection

We invited panellists to participate in the online survey through a personalized e-mail. An assistant not part of the study (CV) assigned a unique number code for each online questionnaire sent. Only CV had access to the linked identity of the panellists and the unique code numbers and managed all e-mail correspondences with the panellists, including the invitation for participation and reminders. The online survey was open for feedback for four weeks; we sent two reminders to non-respondents after two weeks and one week, respectively.

### Ethics

Ethics approval was obtained from the Medical Ethics Committee of the University Hospital of Brussels (B.U.N. 1432023000048). The participating Delphi panellists could leave the study at any time for any reason if they wished to do so without any consequences. All potential professional expert panellists were provided with an information sheet explaining the goals of the study at every round, combined with the information regarding informed consent. The participating panellists were informed about how their opinions will be used within the present study. They were also informed that they could withdraw from the study at any time without any consequences for them. We emphasized in all documents sent to them that only the management assistant (CV) had access to the participant list, and that the core author group members could not know or trace whether or not they participate in the study. In the core author group meetings in Delphi round 1, consent is implied when the core author group voluntarily shared their opinions and knowledge. In Delphi round 2, the informed consent form was added in the first page of the online questionnaire, meaning that they can only complete the questionnaire after their consent to participation.

### Statistics

We analyzed the quantitative data in IBM SPSS statistics 26 (©IBM Corporation) and used MS Excel version 16 (©Microsoft 2022) for qualitative data. We used descriptive statistics to analyze the characteristics of the Delphi panellists. To analyze the level of consensus, we calculated the mean scores for the domains and the central tendencies and dispersion for the recommendations using descriptive statistics. We calculated means, SDs, median, IQR, percent agreement of the 1–5 and 1–10 scales.

### Qualitative data analyses

LVDB and KDN analyzed the qualitative data on the key domains and recommendations independently from each other. Both researchers coded the data according to predefined categories, i.e., suggested changes in the content and/or language of the key domains and key recommendations. Any discrepancy was discussed and resolved with RM. The revised list of domains and recommendations was then sent to the core author group for review and feedback.

### Role of funding source

RM and KDN have received postdoctoral fellowship grants from the Research Foundation Flanders – Fonds Wetenschappelijk Onderzoek (FWO) (grant numbers: 12D4523N and 12AEO24N). The funder had no role in the study design, data collection, data analyses, interpretation or writing of report. Views and opinions expressed are those of the author(s) only and do not reflect those of the Research Foundation Flanders – FWO. The Research Foundation Flanders – FWO cannot be held responsible for them.

## Results

**Fig. 2:**
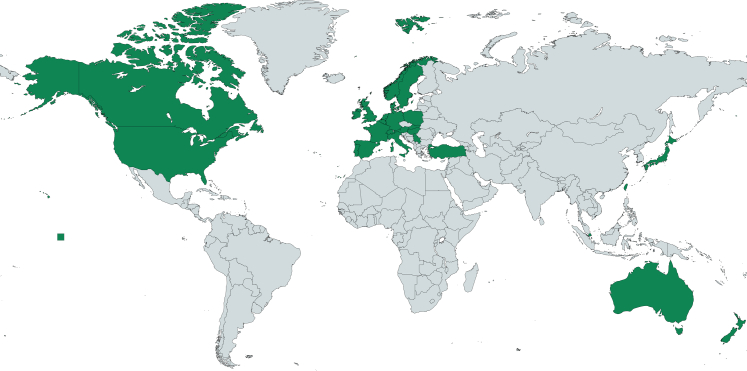
**Geographical location of the Delphi panellists^a^ and the core author group^b^**. The geographical figure was created via Mapchart.net. ^a^The Delphi panellists are located in 28 countries (colored in green): Australia (n = 4), Austria (n = 2), Belgium (n = 9), Canada (n = 3), Denmark (n = 2), England UK (n = 16), France (n = 3), Germany (n = 3), Hungary (n = 1), Ireland (n = 2), Italy (n = 3), Japan (n = 1), Luxemburg (n = 1), Netherlands (n = 5), New Zealand (n = 1), Norway (n = 4), Poland (n = 2), Portugal (n = 1), Scotland UK (n = 1), Serbia (n = 1), Singapore (n = 2), Slovak Republic (n = 1), Spain (n = 4), Sweden (n = 2), Taiwan (n = 1), Turkey (n = 1), United States of America (n = 3), Multiple locations (n = 2). ^b^The core author group members are located in six countries: Belgium (n = 5), England (n = 2), Poland (n = 1), Portugal (n = 1), Switzerland (n = 2).

In total, 82 Delphi panellists (70.1% response rate) from 28 countries in Asia, Australia, Europe, and North America participated in the first online survey (Table 2 and Fig. 2). Of the 93 invited professional experts and 24 older person's representatives, 63 (68%) and 19 (79.2%) respectively participated. Mean age among all Delphi panellists was 54.2 years (SD = 13.6), and 61.0% of them (n = 50) was female. Almost all (n = 81; 98.8%) had post-secondary school education. In terms of specialty, three quarters indicated to have knowledge and experience in palliative care/support for older people, and those living with frailty; almost half in geriatrics; and one third in rehabilitation. About three in four was involved in the topic through research, practice and education; about a third through policymaking. Among participating practitioners (n = 62), most were doctors (n = 40), followed by nurses (n = 12).

**Table 2:** Characteristics of Delphi panellists.

	Total Delphi panellists, n = 82	Professional expert, n = 63	Older person representatives, n = 19
**Age**, mean number in years, SD and range	Mean = 54.2, SD = 13.6, range = 29–83	Mean = 51.6, SD = 10.2, Range = 30–76	Mean = 62.7, SD = 19.1, range = 29–83^a^
**Female gender**, n (%)	50 (61.0)	37 (58.7)	13 (68.4)
**Highest educational attainment**, n (%)			
No education and Primary school education	0	0	0
Secondary school education	1 (1.2)	0	1 (5.3)
Post-secondary school education	81 (98.8)	63 (100.0)	18 (94.7)
**Specialties in which they felt they have experience/knowledge**,^b^ n (%)			
Palliative care and support for older people	64 (78.0)	54 (85.7)	10 (52.6)
Geriatric care and support for older people	56 (68.3)	46 (73.0)	10 (52.6)
Rehabilitative care and support for older people	29 (35.4)	21 (33.3)	8 (42.1)
Palliative care and support for older people with frailty	63 (76.8)	51 (81.0)	12 (63.2)
Geriatric care and support for older people with frailty	46 (56.1)	31 (61.9)	7 (36.8)
Rehabilitative care and support for older people with frailty	26 (31.7)	22 (34.9)	4 (21.1)
No experience/knowledge of the abovementioned topics	4 (4.9)	1 (1.6)	3 (15.8)
**Obtained their experience or knowledge through**,^b^^,^^c^ n (%)			
Research	60 (76.9)	52 (83.9)	8 (50.0)
Education	54 (69.2)	45 (72.6)	9 (56.3)
Policy	23 (29.5)	18 (29.0)	5 (31.3)
Practice	58 (74.4)	47 (75.8)	11 (68.8)
**Current professional background**,^d^ n			
**Researcher**^e^	**28**	**25**	**3**
**Policy-making**	**2**	**–**	**2**
**Practitioner**	**62**	**52**	**10**
Medicine^f^	40	37	3
Nursing	12	9	3
Social work	5	1	4
Physical therapy	3	3	0
Occupational therapy	1	1	0
Psychology	2	2	0
Ethics	1	1	0
**Other**^g^	**6**	**0**	**6**

a Older persons' representatives who are older than 65 years and older (n = 11).

b Multiple answers were possible. These are Delphi panellists' perception of their experience and knowledge.

c Four of the Delphi panellists felt that they had no experience nor knowledge on the abovementioned topics.

d Fourteen experts and two representatives have combined professions in research, practice and/or policy, e.g., nurse scientist, psychologist/researcher, physician and health services research, nurse, and university professor.

e Research-related professions: expert = university professor, postdoctoral researcher, researcher, gerontologist, epidemiologist | older person's representatives = university professor.

f Examples of medical specialties: geriatrics (n = 8), palliative medicine (n = 3), internal medicine (n = 3), general practice (n = 2).

g Other professional background: older person's representatives = trade union officer, project manager, consultant human resource, telecom manager, organization president, and chartered surveyor.

Through iterative discussions in Delphi round 1, the authors identified 11 key domains that jointly define an integrative palliative, geriatric, and rehabilitative approach to care and support for older people living with frailty and their family carers. For each domain, we identified one or more recommendations, with a total of 34. Appendix 4 explains the major considerations for the development of this first list.

In Delphi round 2, the panellists immediately reached full consensus on all domains (Table 3) and recommendations (Table 4). The total mean importance scores ranged from 8.9 for domain 10 “*community and public health approaches*” to 9.6 for domain 2 “*holistic person-centered care and support focused on both capacities and needs*”, domain 4 “*communication and shared decision-making*”, and domain 5 “*optimal care and dying with comfort and dignity*” (Table 3). The panellists reached very high agreement on 21 key recommendations and high agreement on 13 key recommendations (Table 4).

**Table 3:** Level of consensus regarding the importance of key domains.

^a^Processing of scores: Full consensus = mean score of 8 or higher | Moderate consensus = mean score between 6 and 8 | no consensus = mean score of 6 or lower.

**Table 4:** Level of consensus on their agreement for each preliminary recommendation and domain.

Preliminary list of key domains and recommendations^a^	Total Delphi panellists, n = 82	Professional experts, n = 63	Older person representatives, n = 19
**DOMAIN 1: Applicability of palliative, geriatric, and rehabilitative care approach in frailty**			
Recommendation 1.1	Median: 5 IQR: 0 % score 4 or 5: 93.9%	Median: 5 IQR: 0 % score 4 or 5: 93.6%	Median: 5 IQR: 0 % score 4 or 5: 94.7%
Recommendation 1.2	Median: 5 IQR: 0 % score 4 or 5: 96.3%	Median: 5 IQR: 0 % score 4 or 5: 95.1%	Median: 5 IQR: 0 % score 4 or 5: 100%
Recommendation 1.3	Median: 5 IQR: 1 % score 4 or 5: 90.0%	Median: 5 IQR: 1 % score 4 or 5: 90.2%	Median: 5 IQR: 0 % score 4 or 5: 88.9%
**DOMAIN 2: Holistic person-centered care and support focused on both capacities and needs**			
Recommendation 2.1	Median: 5 IQR: 0 % score 4 or 5: 97.5%	Median: 5 IQR: 0 % score 4 or 5: 96.6%	Median: 5 IQR: 0 % score 4 or 5: 100%
Recommendation 2.2	Median: 5 IQR: 0 % score 4 or 5: 98.7%	Median: 5 IQR: 0 % score 4 or 5: 98.3%	Median: 5 IQR: 0 % score 4 or 5: 100%
**DOMAIN 3: Goal-oriented and pro-active care and support**			
Recommendation 3.1	Median: 5 IQR: 1 % score 4 or 5: 97.4%	Median: 5 IQR: 1 % score 4 or 5: 96.6%	Median: 5 IQR: 0 % score 4 or 5: 100%
Recommendation 3.2	Median: 5 IQR: 0 % score 4 or 5: 93.6	Median: 5 IQR: 0 % score 4 or 5: 91.6%	Median: 5 IQR: 1 % score 4 or 5: 100%
Recommendation 3.3	Median: 5 IQR: 1 % score 4 or 5: 93.6%	Median: 5 IQR: 1 % score 4 or 5: 93.2%	Median: 5 IQR: 1 % score 4 or 5: 94.5%
Recommendation 3.4	Median: 5 IQR: 0 % score 4 or 5: 96.2%	Median: 5 IQR: 0 % score 4 or 5: 96.7%	Median: 4.5 IQR: 1 % score 4 or 5: 94.4% (Moderate consensus)
Recommendation 3.5	Median: 5 IQR: 0 % score 4 or 5: 98.7%	Median: 5 IQR: 0 % score 4 or 5: 98.3%	Median: 5 IQR: 0 % score 4 or 5: 100%
Recommendation 3.6	Median: 5 IQR: 0 % score 4 or 5: 100%	Median: 5 IQR: 0 % score 4 or 5: 100%	Median: 5 IQR: 0 % score 4 or 5: 100%
**DOMAIN 4: Communication and shared decision-making**			
Recommendation 4.1	Median: 5 IQR: 0 % score 4 or 5: 98.7%	Median: 5 IQR: 0 % score 4 or 5: 98.3%	Median: 5 IQR: 0 % score 4 or 5: 100%
Recommendation 4.2	Median: 5 IQR: 0 % score 4 or 5: 97.4%	Median: 5 IQR: 0 % score 4 or 5: 98.3%	Median: 5 IQR: 0 % score 4 or 5: 94.5%
**DOMAIN 5: High-quality end-of-life care and dying with comfort and dignity**			
Recommendation 5.1	Median: 5 IQR: 0 % score 4 or 5: 97.4%	Median: 5 IQR: 1 % score 4 or 5: 98.3%	Median: 5 IQR: 0 % score 4 or 5: 94.4%
Recommendation 5.2	Median: 5 IQR: 0 % score 4 or 5: 93.5%	Median: 5 IQR: 1 % score 4 or 5: 93.1%	Median: 5 IQR: 0 % score 4 or 5: 94.4%
Recommendation 5.3	Median: 5 IQR: 1 % score 4 or 5: 93.5%	Median: 5 IQR: 1 % score 4 or 5: 91.4%	Median: 5 IQR: 0 % score 4 or 5: 100%
**DOMAIN 6: Family or informal caregivers (if applicable) as provider and recipient of care and support**			
Recommendation 6.1	Median: 5 IQR: 0 % score 4 or 5: 100%	Median: 5 IQR: 0 % score 4 or 5: 100%	Median: 5 IQR: 0 % score 4 or 5: 100%
Recommendation 6.2	Median: 5 IQR: 0 % score 4 or 5: 94.8%	Median: 5 IQR: 0 % score 4 or 5: 93.1%	Median: 5 IQR: 0 % score 4 or 5: 100%
Recommendation 6.3	Median: 5 IQR: 1 % score 4 or 5: 97.4%	Median: 5 IQR: 1 % score 4 or 5: 98.3%	Median: 5 IQR: 0 % score 4 or 5: 94.5%
**DOMAIN 7: Integrated interdisciplinary care and support, and access to services**			
Recommendation 7.1	Median: 5 IQR: 0 % score 4 or 5: 98.7%	Median: 5 IQR: 0 % score 4 or 5: 98.3%	Median: 5 IQR: 0 % score 4 or 5: 100%
Recommendation 7.2	Median: 5 IQR: 1 % score 4 or 5: 96.1%	Median: 5 IQR: 1 % score 4 or 5: 94.8%	Median: 5 IQR: 0 % score 4 or 5: 100%
Recommendation 7.3	Median: 5 IQR: 1 % score 4 or 5: 87.0%	Median: 5 IQR: 1 % score 4 or 5: 82.7%	Median: 5 IQR: 1 % score 4 or 5: 100%
**DOMAIN 8: Care and support by competent professionals**			
Recommendation 8.1	Median: 5 IQR: 0 % score 4 or 5: 93.5%	Median: 5 IQR: 0 % score 4 or 5: 91.4%	Median: 5 IQR: 0 % score 4 or 5: 100%
Recommendation 8.2	Median: 5 IQR: 1 % score 4 or 5: 92.2%	Median: 5 IQR:1 % score 4 or 5: 89.6%	Median: 5 IQR: 1 % score 4 or 5: 100%
Recommendation 8.3	Median: 5 IQR: 0 % score 4 or 5: 96.1%	Median: 5 IQR: 0 % score 4 or 5: 94.9%	Median: 5 IQR: 0 % score 4 or 5: 100%
**DOMAIN 9: Contextualized and culture-sensitive care and support**			
Recommendation 9.1	Median: 5 IQR: 1 % score 4 or 5: 92.2%	Median: 5 IQR: 0 % score 4 or 5: 91.4%	Median: 5 IQR: 1 % score 4 or 5: 94.5%
Recommendation 9.2	Median: 5 IQR: 0 % score 4 or 5: 96.1%	Median: 5 IQR: 0 % score 4 or 5: 94.9%	Median: 5 IQR: 0 % score 4 or 5: 100%
Recommendation 9.3	Median: 5 IQR: 1 % score 4 or 5: 87.0%	Median: 5 IQR: 1 % score 4 or 5: 86.2%	Median: 5 IQR: 1 % score 4 or 5: 88.9%
**DOMAIN 10: Community and public health or health promotion approaches**			
Recommendation 10.1	Median: 5 IQR: 1 % score 4 or 5: 93.5%	Median: 5 IQR: 1 % score 4 or 5: 93.1%	Median: 5 IQR: 1 % score 4 or 5: 94.4%
Recommendation 10.2	Median: 5 IQR: 1 % score 4 or 5: 92.2%	Median: 5 IQR:1 % score 4 or 5: 89.7%	Median: 5 IQR: 0 % score 4 or 5: 100%
Recommendation 10.3	Median: 5 IQR: 0 % score 4 or 5: 87.0%	Median: 5 IQR: 1 % score 4 or 5: 84.5%	Median: 5 IQR: 0 % score 4 or 5: 94.5%
**DOMAIN 11: Ethical principles and frameworks**			
Recommendation 11.1	Median: 5 IQR: 0 % score 4 or 5: 93.5%	Median: 5 IQR: 1 % score 4 or 5: 91.3%	Median: 5 IQR: 0 % score 4 or 5: 100%
Recommendation 11.2	Median: 5 IQR: 0 % score 4 or 5: 96.1%	Median: 5 IQR: 0 % score 4 or 5: 94.8%	Median: 5 IQR: 0 % score 4 or 5: 100%
Recommendation 11.3	Median: 5 IQR: 1 % score 4 or 5: 90.8%	Median: 5 IQR: 1 % score 4 or 5: 91.3%	Median: 5 IQR: 0 % score 4 or 5: 88.3%

Missing data, n: Older person representatives [recommendations 1.2–11.1 = 1] [recommendation 11.2–11.3 = 2] | Professional experts [recommendation 1.3–3.6 = 3] [recommendations 4.1–11.3 = 4].

a Processing of scores: Full consensus (very high agreement = median of 5 AND IQR = 0, AND ≥ 80% scoring of 4 or 5; high agreement = median of 5 AND IQR ≤ 1, AND ≥ 80% scoring of 4 or 5) | Moderate consensus (moderate agreement = median 4–5 AND IQR ≤ 2, AND ≥ 60% scoring of 4 or 5; low agreement = median 4–5 AND IQR ≤ 2 OR ≥ 60% scoring of 4 or 5) | No consensus (no agreement = Median between 2 and 4; high disagreement = median 1 AND IQR = 0 AND 80% scoring of 1 or 2).

Of all panellists, 54 professional experts and 16 older person's representatives provided in varying extent qualitative comments on the domains and recommendations. Based on these qualitative data, minor adaptations were made to improve language without changing meaning, to clarify terms where needed, and to abbreviate or split sentences for better readability. Cross-cutting adaptations concerned the consistent use of ‘older person or older people living with frailty’ to stress the importance of the person behind the patient who is living with frailty; and ‘family carers’ instead of family, informal carer or other terms used in the literature.

Some panellists confirmed the need for an integrative framework, e.g., a professional expert wrote, “*[…] these clusters of categories [referring to the disciplines] are maybe helpful for practitioners and systems of care but for the person the distinctions are arbitrary*”. Some panellists commented on the overlap between domains e.g., a professional expert explained, “*All domains are very and equally important, and an overlap can be seen (which is not wrong)*”. Some mentioned finding it difficult to evaluate the importance of the domains, explaining that all domains are important, desirable, and difficult to disagree with. Others also mentioned that the importance of some of the domains and the three disciplines might vary from one person to another or depending on the stage/severity of frailty. For example, older person's representatives mentioned: “*Depending on the care need(s) and the patient's wishes concerning their needs, one of the three [disciplines] will have the most emphasis*”; “*[…] the weight of each specialty will vary according to the person and to the evolution of her frailty. Integrated approach will reinforce efficiency of the global approach, each specialty supporting the action of the others*”. A professional expert said: “*The principles of geriatric and rehabilitative approach are more important at lower levels of frailty*”.

Some panellists argued that domains 9, 10, and 11 concerning the societal and public health perspectives might not fit in this work and deemed it better to concentrate on a clinical approach to care and support. However, others praised the broad perspective taken in this work, which bridges clinical, health service, and public health perspectives. One professional expert wrote: “*The recommendations convey very much dual perspectives of social and health sciences. This is vital for this population group to move beyond biomedical model of aging to encompass impact of social and societal perspectives*”.

Although consensus was reached immediately in the first online survey, there were a few dissenting voices regarding the applicability of palliative care in frailty. A professional expert wrote: “*Making this association can be very dangerous, since it can prevent certain measures that could be beneficial for the person from being taken*” and “*I do not agree that the approach to the fragile patient should be done from palliative care. It must be geriatrician who does it. The frail patient is not a patient with life-threatening illness*”. This expert also added that depending on the definition of frailty, a person is no longer frail if they are dying, and considers dying and being frail as separate non-overlapping categories. Another professional expert wrote: “*I think nobody will disagree with this (referring to recommendation 1.2), but ageism is a threat. By using this recommendation, the public might understand ALL older people need this care, and as a result might consider them as less autonomous and more dependent than is actually the case*”. There was also a dissenting voice from a professional expert regarding the association between palliative care and rehabilitation, stating that “*[…] palliative and rehabilitative care should not be combined. They are responses to different goals of care*”. One professional expert questioned the order of the disciplines in the integrative approach, stating that palliative care should come last as this is linked to death, while rehabilitation should come first given its focus on recovery.

Finally, we found two recurring comments from panellists. First, many domains and recommendations are not specific for frailty but should generally be a part of care for people with declining health or serious illness, e.g., a professional expert wrote: “*Many of the domains are not specific for frailty but should be integrated in every sort of care*”. Second, while acknowledging the importance of the domains, many panellists highlighted that some of the recommendations lacked concrete tools, guidance, or examples on how they can be implemented in practice.

The final list of domains and recommendations is shown in Table 5. The evidence base for the recommendations, which also highlights the evidence gaps and future priorities, are presented in Appendix 5.

**Table 5:** Final list of key domains and recommendations for this EAPC White Paper on Frailty.^22^^,^^23^

## Discussion

Based on empirical evidence, the input of the EAPC Reference Group on Aging and Palliative Care, and the 82 experts from 28 countries, international consensus was achieved for 11 key domains and 34 recommendations that constitute the very first White Paper defining an integrative palliative, geriatric and rehabilitative approach to care and support for older people living with frailty and their family carers. The integrative approach capitalizes on the strengths of each discipline. Its key recommendations elucidate what is needed from clinical, health service, and public health perspectives to address the multidimensional needs of older people living with frailty and their family carers, to support their capacities and resources, and to maintain or improve their quality of life and well-being until the end of their lives, including bereavement.

Remarkably, we reached consensus immediately in the first online survey, both for professional experts and older person's representatives. This might be explained by the thorough, iterative preparations of the international and interdisciplinary core author group and their strong consideration of the existing empirical evidence in frailty, resulting in a comprehensive and carefully formulated list of domains and recommendations in Delphi round 1. Further, we formulated the recommendations in such a manner that they would apply across settings, countries, and health care systems, and include aspects that are important to older persons and all those involved in caring for and supporting them, i.e., family carers, health and social care professionals, volunteers, and communities. The multi-level approach ensured that we could also address issues beyond the traditional clinical perspective but particularly relevant for older people living with frailty in many societies, including workforce issues, human rights, intersectionality, and concerns about ageism or discrimination.^24^, ^25^, ^26^, ^27^ While some panellists commented that not all domains were specific to frailty, as they are applicable for all older people with declining health, we had intentionally chosen to include aspects of generic relevance for completeness and tailored each recommendation to older people living with frailty.

Although palliative care, geriatrics, and rehabilitation are disciplines with own specialized knowledge and unique contributions, our Delphi study showed that their principles, values, goals, and components can be integrated well to jointly create an integrative approach for older people living with frailty and their family carers.^28^ As one of our panellists explained, working in silo might be helpful for a specific group of practitioners or within a system of care, but for a person, distinctions between disciplines are arbitrary. In Domain 1, we argued that the integrative palliative, geriatric, and rehabilitative approach is applicable from diagnosis of frailty until the end of life, although we do acknowledge variations in the balance of each of these disciplines depending on the person's needs and preferences across the frailty trajectory. Our integrative approach reinforces the value and appropriateness of personalized palliative care earlier in the frailty trajectory and acknowledges the added value of personalized rehabilitation throughout the whole frailty trajectory up until death, to enable people living with frailty to live as fully and independently as possible until they die.^29^^,^^30^ However, our qualitative data revealed that not all panellists fully agreed with this. A few professional panellists commented that palliative care as an approach is mainly applicable at the end of life and is not relevant for all people with frailty. Some mentioned that rehabilitation should come first in our integrative approach as it relates to recovery. With this interdisciplinary work, we hope to supersede these persistent misperceptions and make clear the value of integrative palliative, geriatric and rehabilitative care and support throughout the frailty trajectory, especially when personalized to each person, their priorities, needs, and wishes.

Central to our integrative approach is the combined focus on needs and capabilities of older people living with frailty. Whilst palliative care's primary focus has been on addressing multidimensional needs and concerns to improve quality of life near the end of life,^31^ aging disciplines have tended to focus more on people's individual intrinsic capacities or capabilities, their adaptive coping strategies and resources, their resilience and health assets, and how these can help to optimize quality of life and well-being across the frailty trajectory.^32^ Our work brings these two angles together, to ensure an assets-based focus that recognizes the capabilities of each individual as a person in the context of their social networks, alongside their unique multidimensional needs. This dual, balanced focus is important as older people living with frailty are too often characterized in a negative stereotypical manner as being ill, fragile, vulnerable, dependent or a burden, which loses sight of their personal assets that distinguish them as people.^26^ Our combined approach aligns well with the work within WHO's Decade of Healthy Aging, focusing on optimizing people's intrinsic capacity and functional ability as they age.^27^^,^^32^, ^33^, ^34^

Several panellists highlighted that our White Paper expounds the ideal features and desired attributes for an optimal approach; and questioned whether and how the recommendations can be implemented in routine practice in different health systems. Many health systems indeed face substantial constraints in human and financial resources, which limits the capacity of health and social care professionals to provide the needed care and support. Interdisciplinary working models and integrated person-centered care approaches have been advocated for decades but appear hard to achieve in practice.^34^ This also became clear from the evidence reviewed for this White Paper, as we identified a paucity of high-quality empirical evidence for effective and cost-effective strategies and interventions across the different domains and recommendations. As such, our White Paper represents a first critical step in establishing a gold standard for what an integrative palliative, geriatric, and rehabilitative approach needs to look like in this population. How to implement the recommendations represents a considerable challenge as well as an opportunity for health and care systems worldwide. To be able to respond to the increasing demands while upholding the quality of care and support deserved by all, this work calls for a radical shift in health and care provision and serves as a call to action for individuals, communities, health and care systems, policy and decision-makers to actively commit to integrated working; to dismantle silos; to blur boundaries constructively and collaboratively; and to pool assets to realize efficiency at scale.

Therefore, our overarching research recommendation is to improve our evidence base regarding the implementation of integrative palliative, geriatric, and rehabilitative approach to care and support for this population, and specifically invest in developing, evaluating, and comparing new and existing programmes and policies aiming to improve what older people living with frailty and their family carers value.

This Delphi study used multiple cycles of literature review, iterative discussions with a core international interdisciplinary author group, panellists from 28 countries including both professional experts and older person's representatives, and upheld strict ethical procedures. The open text boxes in the Delphi survey were used considerably by many panellists, which provided in-depth qualitative insights that highly complemented our quantitative evaluation.

The study also had limitations. Only 19 older person's representatives participated, compared to 62 professionals; and of the 19 representatives, 11 were 65 years and older. Future work should focus on engaging with older persons who have lived frailty experiences and their family carers using adapted formats and study designs to ensure that their voices are represented. We recruited older person's representatives through organizations representing older people and patients but involving older persons outside of these channels might have brought other insights. Most panellists represented high-income countries, suggesting our work might not be globally applicable. Finally, while a wide range of professions and disciplines were involved, they were not equally represented e.g., general practitioners or community-based staff were less well represented. Notably, several members of the core author group developing the recommendations do work in research or clinical practice in primary care and communities.

This EAPC White Paper defined an integrative palliative, geriatric and rehabilitative approach to care and support for older people living with frailty and their family carers that capitalizes on the strengths of each specialism. This White Paper presents a gold standard for the care and support for older people living with frailty and their family carers. It also calls for a radical shift in healthcare provision to effectively integrate palliative, geriatric and rehabilitative approaches to care and support for this population and represents a first critical step in establishing how to achieve this. Finally, this work should drive future quality improvement efforts, research, and policy, aiming to improve what older people living with frailty and their family carers value.

## Contributors

LVDB, SP, LP, NVDN, CN, KS, SMP, and RM made equal substantial contributions to conceptualizing the design of the study. LVDB, RM, and KDN acquired, had access to, verified, and analyzed the data. LVDB and RM wrote the first draft of the manuscript and critically revised it for important intellectual content. All authors have contributed substantially to interpreting the data from their own expert and disciplinary perspectives; to revising the manuscript critically; and have participated sufficiently in the work to take responsibility for appropriate portions of the content. All authors read and approved the final version of the manuscript, and they accept responsibility to submit this manuscript for publication.

## Data sharing statement

Data are archived in closed access at the institutional repository of the Vrije Universiteit Brussel (VUB), with metadata available. Data cannot be made available due to the lack of participant consent for data sharing beyond the research team.

## Editor note

The Lancet Group takes a neutral position with respect to territorial claims in published maps and institutional affiliations.

## Declaration of interests

The authors declare no intellectual or financial conflicts of interest with regard to the study.
