# Supplementary material for: A European Association for Palliative Care White Paper defining an integrative palliative, geriatric, and rehabilitative approach to care and support for older people living with frailty and their family carers: a 28-country Delphi study and recommendations
Source: eClinicalMedicine. 2025 Aug 12;87:103403. doi: 10.1016/j.eclinm.2025.103403 (PMC12362020; doi:10.1016/j.eclinm.2025.103403)
Supplement: Appendix 1 [file mmc1.docx]

**Appendix 1. Details of the iterative procedure for the Delphi round 1**

| **Knowledge Sources** | **Descriptions of the procedures** |
| --- | --- |
| **THREE RAPID LITERATURE REVIEWS**  were conducted to identify relevant empirical evidence until January 2022. The list of literature included book chapters, position papers, and scientific articles, including but not limited to: review studies, and qualitative studies or reviews of qualitative studies investigating the perspectives of older people living with frailty and other key stakeholders (i.e. family carers and healthcare providers. | The empirical evidence was predominantly based on reviews, but for recommendations that lacked empirical evidence, position papers (i.e. guidelines or other synthesis combining evidence with consensus or positions), policy reports, legal documents and clinical experience were used.  In three cycles, we conducted rapid literature reviews in January 2020, May 2020, and January 2022. We conducted database search in PubMed and Google Scholar (from inception up to January 2022). We used keywords related to older people living with frailty (“frailty”, “elderly”, “older people”, “older persons”, “older adults” OR “older population”), in combination with keywords for palliative care (“palliative care”, “end-of-life care”, “terminal care”, “death”, OR “dying”) as we aimed to the find work at the intersection of palliative care, geriatrics and rehabilitation and considered palliative care to be the most demarcating discipline. Additionally, we used citation tracking in Google Scholar of the references lists of the identified studies and searched in an Australian database (caresearch.com.au). The authors (representing all three disciplines) of this study also identified relevant literature. This resulted in 164 publications, comprising systematic reviews, primary studies with various research designs (observational, interventional, and multiple quantitative and qualitative methods), and; other article types such as editorials, clinical guidelines; book chapters; and discussion papers.  We found that over the past decades, there were no publications before our Delphi study, that integrated the existing evidence across the disciplines of geriatrics, rehabilitation and palliative care, or an evidence- and consensus-based integrative framework aiming to overcome the specialty silos. We did find work aimed at integrating palliative care and geriatrics, or palliative care and rehabilitation, usually only on a clinical level. This confirmed to need for an integrative framework, spanning all disciplines, and moving beyond clinical to include health service and public health perspectives.  Using this same rapid literature review methodology, we conducted an additional rapid literature review between September 2024 and February 2025 to identify new empirical evidence to complement the earlier cycles of rapid reviews. These additional rapid reviews further strengthened the explanatory texts for the key recommendations. |
| **SEVEN CORE AUTHOR GROUP MEETINGS**  The summary of the most important topics and important decisions made were based on the minutes of each core author group meetings. | Seven core author group meetings were conducted between 29 April 2020 and 9 May 2022. These group meetings were conducted at strategic timepoints that are iterative with the rapid reviews, small core author group meetings, and core author group input through e-mail. The average duration per meeting was 45 minutes.  **Examples of the most important topics discussed in these meetings:**   - The online survey protocol, including the procedures and the Delphi panellists; the definitions of frailty in the context of aging; the preliminary set of key domains and recommendations; the difficulty of labelling specific care as palliative care, as there is a clear overlap between palliative, geriatrics, and rehabilitation; the integration of geriatrics and rehabilitation into the White Paper; the definitions of frailty, palliative care, geriatrics, and rehabilitation identified in literature; the focus on all care and support providers involved in the care for older people living with frailty; the protocol for the online survey, including the paper-based and online questionnaire; the estimated timeline for the ethics application; the Delphi panellist recruitment via the EAPC Reference Group for Aging and Palliative Care Network. - During these meetings, we also evaluated on several occasions the current expertise in the core author group to ensure that the necessary disciplines for the White Paper are covered. We invited and added two core authors with expertise in general practice/primary care and rehabilitative care. Another expert with expertise in spiritual and existential care for older people was invited to provide input.   **Examples of the most important decisions made during these meetings:**  the need to identify and compile different definitions of frailty from the perspectives of palliative care, geriatrics, and rehabilitation; the issue with labelling is difficult to solve and that we cannot separate these disciplines; the need to focus this White Paper on high-quality care for older people living with frailty from the perspectives of integrative palliative, geriatric, and rehabilitative approach to care and support; to use integrative palliative, geriatric and rehabilitative care for older people living with frailty as the main focus of the White Paper; to add another round of online survey particularly with researchers/clinical researcher to identify research priorities; to focus the White Paper on older people living with frailty and their family carers; to use the concept of “support and care needs” instead of only “care needs”; to perform additional rapid literature review to complement the previous cycles of rapid literature reviews and to look for additional literature focusing on geriatrics and rehabilitation in relation to palliative care for older people; to launch the invitation for the EAPC Reference Group for Aging and Palliative Care Network, from which we planned to recruit professional experts as Delphi panellists. |
| **SMALL CORE AUTHOR GROUP MEETINGS** | At multiple strategic timepoints throughout Delphi round 1, RM and LVDB had several small group meetings to integrate empirical evidence and the inputs from the core author group into the protocol for the Delphi study and the key domains and recommendations. All iterations of all documents were fed-back to the core author group per e-mail for reviewal before finalising them. This procedure is important to ensure that inputs from all core authors, especially those who were not present in the core author group meetings, would be considered. When the key domains and recommendations were finalized, RM and LVDB applied for ethics approval for the online surveys from the Medical Ethics Committee of the University Hospital of Brussels – linked with the Vrije Universiteit Brussel, which is the coordinating institute for this Delphi study. This set of key domains and recommendations was required for the Ethics application, as this was the basis of the questionnaire for the online survey in Delphi round 2. |
| **CORE AUTHOR GROUP INPUT THROUGH E-MAIL** | Important document iterations from the small group meeting were fed back to the core authors by e-mail to obtain any additional input and for review, especially from those who were not able to attend the group meetings. Examples of important inputs per e-mail were: 1) the multiple iterations of the key domains and recommendations that resulted to the set of 11 key domains and 34 key recommendations; and 2) the decision to include representatives of older people and their family carers as Delphi panellists with the corresponding recruitment strategies. |
