# Supplementary material for: A European Association for Palliative Care White Paper defining an integrative palliative, geriatric, and rehabilitative approach to care and support for older people living with frailty and their family carers: a 28-country Delphi study and recommendations
Source: eClinicalMedicine. 2025 Aug 12;87:103403. doi: 10.1016/j.eclinm.2025.103403 (PMC12362020; doi:10.1016/j.eclinm.2025.103403)
Supplement: Appendix 2 [file mmc2.docx]

**Appendix 2. The questionnaire for the first online survey in Delphi round 2**

| **Questionnaire development** | |
| --- | --- |
| The preliminary list of key domains and recommendations was used to develop a paper-based questionnaire for the online survey in Delphi round 2. This questionnaire collected quantitative and qualitative data on the panellist characteristics and the evaluation of the key domains and recommendations. It was piloted for length, clarity and overall adequacy with five peer health and social science researchers. Necessary amendments were made to clarify ambiguities and to improve the quality and feasibility of the survey. The developed paper-based questionnaire was made available in LimeSurvey (@LimeSurvey GMBH), which was piloted using the same procedure above. | |
| **Main concepts** | **Survey questions** |
| **Delphi panellist characteristics** | - **What is your age in years?** - **What is your gender? Options included male, female, prefer not to say, other please specify.** - **What is your highest educational attainment? Options included no education, primary education, secondary education, post-secondary education** - **Do you feel like you have experience or knowledge in**(please click all that apply). Options included palliative care, geriatric care, rehabilitative care, both for older people and older people with frailty; no experience and knowledge - **Follow-up question for those who answered that they do have such experience and knowledge: Did you get this experience or knowledge through** (please click all the apply): Options included research, education, practice and policy, other - **Which country are you from?** - **In which country are you now residing?** - **What is/was your profession?** |
| **Evaluation of key domains**   - using a 10-point importance scale | **Quantitative evaluation of each of the key domains:** Please rate how important the following key domains are as part of an optimal approach to care and support for older people living with frailty on a 10-point importance scale (**1 = not important to 10 = very important)**:  **Domain 1: Applicability of palliative, geriatric, and rehabilitative care approach in frailty**  **Domain 2: Holistic person-centred care and support focused on both capacities and needs**  **Domain 3: Goal-oriented and pro-active care and support**  **Domain 4. Communication and shared decision-making**  **Domain 5: Optimal end-of-life care and dying with comfort and dignity**  **Domain 6: Family as provider and recipient of care and support**  **Domain 7: Integrated interdisciplinary care and support, and access to services**  **Domain 8: Care and support by competent professionals**  **Domain 9: Contextualised and culture-sensitive care and support**  **Domain 10: Community and public health approaches**  **Domain 11: Ethical principles and frameworks** |
|  | **General qualitative comment: Do you have feedback on the identified domains for an optimal approach to care and support for older people living with frailty, such** as comments/suggestions (e.g. rephrasing or using other words), explanations of your ratings or ideas for other domain that is not in the list? |
| **Evaluation of key recommendations (see preliminary list of recommendations hereunder)** | **Quantitative evaluation of each recommendation:** Please rate your level of agreement for each key recommendation (using a five-point agreement scale). |
|  | **Qualitative comment for each key recommendation:** Explanation for rating, or any comments/suggestions, such as rephrasing or reformulating texts |
|  | **Overall qualitative comment:** Do you have any other comments about this survey, such as ideas for key domains and recommendations that we have not included in the list? Your feedback can help us improve the next survey round. |
| **Domain 1: Applicability of palliative, geriatric, and rehabilitative care approach in frailty**  **Recommendation 1.1**  An optimal approach for older people living with frailty should be an integrative palliative, geriatric, and rehabilitative approach, combining the principles or approaches developed in all three disciplines, and centred around the person with frailty.  **Recommendation 1.2**  The integrative palliative, geriatric, and rehabilitative approach is in principle applicable throughout the frailty trajectory, albeit there might be a varying emphasis on the specificities of each of three disciplines depending on the needs and preferences, of the person across the frailty trajectory.  **Recommendation 1.3**  An integrative palliative, geriatric, and rehabilitative approach for older people with frailty and their family is applicable across all care and support settings and services. This includes (acute and community) hospital care, outpatient and ambulatory care, hospice care, intermediate care, care at home and care in nursing or care homes (long term care facilities), and also applies during transitions between settings. | |
| **Domain 2: Holistic person-centred care and support focused on both capacities and needs Recommendation 2.1**  Care and support for older people with frailty should be person-centred and tailored to the older person’s needs; these can be functional, physical, or medical, as well as psychological, spiritual or existential, social, or practical. Needs assessment should be comprehensive, multidimensional, and holistic, and revisited on a regular basis. As many frail older people develop multiple conditions throughout their trajectory, identifying the cumulative effects of multiple co-existing conditions is necessary.  **Recommendation 2.2**  Complementary to a focus on needs, it is important to promote quality of life and well-being by focusing on empowerment and enabling intrinsic capacities. This implies to not only focus on weaknesses or deficits of older people living with frailty, but also on their strengths and assets, and supporting those to the fullest. | |
| **Domain 3:** **Goal-oriented and pro-active care and support**  **Recommendation 3.1**  Care for older people living with frailty should be goal-oriented and based on people’s priorities, personal preferences, and underlying values i.e. on what matters to them. This includes the setting of realistic and attainable goals jointly with the person and regular re-evaluation of them. **Recommendation 3.2**  Planning of care and support for older people living with frailty should be pro-active instead of reactive alone, and this throughout the whole trajectory. Although the timing of decline or death is difficult to predict, indicators of deterioration or change should be timely recognized. Both pro-active as well as good reactive care are needed – crisis and unexpected events will happen, so it should be both (although currently we lack the pro-active more than the reactive).  **Recommendation 3.3**  Older people living with frailty should be offered the opportunity to discuss advance care planning (ACP). ACP practices should follow up-to-date evidence-based guidelines and recommendations. These highlight the importance of an ongoing process of reflection and communication focused on values, goals and preferences for future treatment and care, and the involvement of family (if present), next to health care providers. While a sole focus on advance directives should be avoided, ACP encourages people to identify a personal representative and to record and regularly review their preferences.  **Recommendation 3.4**  It is important to acknowledge clinical uncertainty as an inherent feature of a frailty trajectory and engage in parallel planning of care. Parallel care planning means to consider a range of potential outcome options for care (e.g. simultaneous parallel planning for recovery and deterioration in health), to offer to discuss this with patients (and families, if present), and plan care in parallel with them.  **Recommendation 3.5**  Emergency response and out-of-hours planning (‘what to do in case of an emergency or out of hours’) should be an important part of a pro-active care plan to support older people living with frailty, their family, and involved professionals.  **Recommendation 3.6**  Care for older people living with frailty can include Interventions such as medication review and reducing inadequate polypharmacy to ensure people are only taking medications that are likely to be beneficial for them. Such interventions aim to support people to remain well and independent for as long as possible and reduce the risk of ill health and poor outcomes. | |
| **Domain 4: Communication and shared decision-making**  **Recommendation 4.1**  Person-centred communication and shared decision-making are crucial components of care for older people living with frailty and their family. This includes sensitive, open, empathetic, and tailored information and communication, which invites people to participate in shared decision-making, while avoiding stereotyping, prejudicial or discriminatory language.  **Recommendation 4.2**  As capacity is decision-, time-, and context-specific, it is important to tailor communication with older people living with frailty with diminishing cognitive and decisional capacities to the person. People should be supported in conversations as much as possible, by using easy-to-read-and-understand materials or creating adequate contexts. | |
| **Domain 5: High-quality end-of-life care and dying with comfort and dignity**  **Recommendation 5.1**  It is important to recognize when an older person with frailty may be dying, to understand the process of dying, to sensitively communicate with patients and families about dying (tailored to a person’s information preferences), and to provide care for dying people using best-practice guidelines. This will enable the delivery of high-quality end-of-life care and dying with comfort, through effective monitoring and assessment of the person’s condition and symptoms over time, the provision of specialist palliative care (counselling) if needed, and support for families throughout and after the dying process.  **Recommendation 5.2**  As the death of many older people who lived with frailty is preceded by one or several medical end-of-life decisions evidence-based and careful decision-making and communication about such decisions with patients and their families is needed.  **Recommendation 5.3**  To enable dying well among older people living with frailty, it is important to communicate sensitively (following people’s information preferences) with people about preferences and possibilities on where and how they would prefer to live and die, what a dignified end-of-life means to them, and enable them, as much as possible, to die following their preference and choice. | |
| **Domain 6:** **Family or informal caregivers (if applicable) as provider and recipient of care and support**  **Recommendation 6.1**  Family or informal caregivers of older persons living with frailty can be relatives, but also close friends, befriended professionals, neighbours or others important to the older person. If available, they often play a crucial role in providing care, rehabilitation, and tangible support to older people living with frailty, in different places of care. Therefore, they should be considered as potential partners in the care planning and decision-making process with whom to build joint partnerships, as preferred by the older person and themselves.  **Recommendation 6.2**  Next to providing care, family or informal caregivers should be recognized as potential care recipients as they might at times become overburdened and in need of support for themselves. Tailored support, training or education for family/informal caregivers should be focused on their needs as well as on empowering their intrinsic capacities and supporting their adaptive coping strategies.  **Recommendation 6.3**  There should be attention to anticipatory grief, and after-death bereavement support for family/informal caregivers, including involving community support initiatives and specialist mental health support if needed. | |
| **Domain 7: Integrated interdisciplinary care and support, and access to services**  **Recommendation 7.1**  An integrated care approach with attention to ensuring coordination and continuity of care for older people living with frailty and their family is needed. This should be centred around the needs of older persons, their families, and their communities. Particular attention is needed to integrate health care, social care, community and voluntary support, to optimizing communication and collaboration when people move between settings or caregivers.  **Recommendation 7.2**  Interdisciplinary team-based collaboration (i.e. working collaboratively and using shared guidelines and protocols) is needed, with those working from different professions and sectors delivering services in a collaborative way with the older people with frailty and their family at the centre. This should result in a personalized and shared care planning process in which goals are set and decisions are made collaboratively. People who can support this process include case managers, care coordinators, care navigators, or link workers.  **Recommendation 7.3**  Timely and needs-based access to health and social care or support services for all older people living with frailty is important, and particularly to rehabilitation, and to social and palliative care services as referral is often too slow and too late. New models of care such as short-term integrated palliative care models for older people are promising, as are community-based support interventions to increase timely access to services. Next to age, other social and structural determinants of health impacting access need consideration. | |
| **Domain 8: Care and support by competent professionals**  **Recommendation 8.1**  Professionals who care for and support older people living with frailty and their family should develop generalist palliative, geriatric medicine and rehabilitative care competencies through under- and postgraduate education and training, as part of a life-long learning trajectory. **Recommendation 8.2**  In all places where older people with frailty live, continuous quality improvement initiatives should be set up to ensure high-quality evidence-based care and support are provided. **Recommendation 8.3**  The workforce of health and social care professionals who provide care for and support older people living and dying with frailty and their families should be resilient, qualified, and respected. Support for them should be available as needed. | |
| **Domain 9: Contextualised and culture-sensitive care and support**  **Recommendation 9.1**  An integrative palliative, geriatric and rehabilitative approach should be delivered in any type of health care system or setting, specialist or non-specialist, in various health care systems and contexts.  **Recommendation 9.2**  Culturally appropriate, culturally competent, and culturally sensitive care provision is important for all older people living with frailty and their families, regardless of their age, gender, social situation, background, ethnicity, language, religion, spirituality, nationality, origin or other characteristics. It implies professionals strive to work within the cultural contexts of the persons they care for, a process which involves the integration of cultural awareness, knowledge, skills, and sensitivity.  **Recommendation 9.3**  An integrative palliative, geriatric and rehabilitative approach should always be provided with respect for patient and human rights. | |
| **Domain 10: Community and public health or health promotion approaches**  **Recommendation 10.1**  Community initiatives and public health or health promotion approaches should be promoted, including compassionate, caring, or healthy aging communities or neighbourhoods embracing aging and dying as normal social parts of life.  **Recommendation 10.2**  Communities and volunteers play an important role in supporting older people living with frailty, as do families and professionals, and should receive adequate resources, support, tailored training and respect.  **Recommendation 10.3**  Older people living in frailty, and their family caregivers, should be valued in society throughout their life, and social exclusion should be prevented. | |
| **Domain 11: Ethical principles and frameworks**  **Recommendation 11.1**  It is important to reject stereotyping and discrimination based on old age as they lead to increased vulnerability. Ageism – a social construct referring to the systematic negative and stereotypical portrayal of older people in society – should be prevented via raising awareness, age-appropriate language, and policies, and supporting initiatives that make older people visible in society in a realistic way, including those who are frail and need support.  **Recommendation 11.2**  All end-of-life decision-making should put the person living with frailty at the centre, preventing over- as well as under-treatment, and ensuring appropriate and proportionate care, treatment, and support, in accordance with preferences and values.  **Recommendation 11.3**  In times of disruptive events such as disaster, pandemic or war, care should remain based on ethical principles and frameworks. Therefore, regular monitoring, reflection, and adaptations of practice or policy are needed before, during and after such crisis periods. | |
