# Supplementary material for: A European Association for Palliative Care White Paper defining an integrative palliative, geriatric, and rehabilitative approach to care and support for older people living with frailty and their family carers: a 28-country Delphi study and recommendations
Source: eClinicalMedicine. 2025 Aug 12;87:103403. doi: 10.1016/j.eclinm.2025.103403 (PMC12362020; doi:10.1016/j.eclinm.2025.103403)
Supplement: Appendix 3 [file mmc3.docx]

**Appendix 3. Processing of scores to determine the level of consensus among Delphi panellists**

|  | **Full consensus** | **Moderate consensus** | **No consensus** | **Action** |
| --- | --- | --- | --- | --- |
| **KEY DOMAINS^a^** |  |  |  |  |
| - Mean score of 8 or higher | X |  |  | To be included in the White Paper |
| - Mean score between 6 and 8 |  | X |  | May be revised; next Delphi round |
| - Mean score of 6 or lower |  |  | X | To be eliminated |
| **RECOMMENDATIONS^b^** |  |  |  |  |
| - Median of 5 AND IQR=0, AND ≥ 80% scoring of 4 or 5 - **Very high agreement** | X |  |  | To be included in the White Paper |
| - Median of 5 AND IQR ≤1, AND ≥ 80% scoring of 4 or 5 - **High agreement** | X |  |  | To be included in the White Paper |
| - Median 4-5 AND IQR ≤2, AND ≥ 60% scoring of 4 or 5 - **Moderate agreement** |  | X |  | May be revised;  for next Delphi round |
| - Median 4-5 AND IQR ≤2 OR ≥ 60% scoring of 4 or 5 - **Low agreement** |  | X |  | May be revised;  for next Delphi round |
| - Median between 2 and 4 - **No agreement** |  |  | X | To be eliminated |
| - Median 1 AND IQR=0 AND 80% scoring of 1 or 2 - **High disagreement** |  |  | X | To be eliminated |

^a^ Evaluation of the key domains on a 10-point importance scale, where 1=not important and 10=very important.

^b^ Evaluation of the key recommendations and the recommendations for future research priorities on a 5-point agreement scale: Strongly disagree (1); moderately disagree (2); neither agree nor disagree (3); moderately agree (4); and strongly agree (5).
