# Supplementary material for: A European Association for Palliative Care White Paper defining an integrative palliative, geriatric, and rehabilitative approach to care and support for older people living with frailty and their family carers: a 28-country Delphi study and recommendations
Source: eClinicalMedicine. 2025 Aug 12;87:103403. doi: 10.1016/j.eclinm.2025.103403 (PMC12362020; doi:10.1016/j.eclinm.2025.103403)
Supplement: Appendix 4 [file mmc4.docx]

**Appendix 4. Key considerations of the core author group in Delphi round 1**

| **1** | We explicitly chose to cover domains spanning clinical, health service, and public health levels, as frailty has important implications at all these levels. |
| --- | --- |
| **2** | We decided to define our population as ‘older people living with frailty’ without strictly mentioning chronological age. Contrary to age cut-offs such as 65+ or 70+ that are being used internationally, we chose to highlight the importance of biological over chronological age and considered strict age cut-offs as possibly discriminatory or ageist. |
| **3** | We decided to focus on how we should care for or support older people living with frailty and their family carers and not on how to screen for or identify those living with frailty. We do recognize the importance of early identification of frailty for effective management and would like to refer to most recent literature on this.(3,4) |
| **4** | We defined family carers broadly to include family members, close friends, befriended professionals, neighbours or others who are in a close supportive or caring role, have strong emotional and social bonds with, and share in the illness experience of the person living with frailty.(18,19) |
| **5** | We made deliberate choices regarding the use of certain terminologies i.e.   1. the development of an ‘integrative’ approach that highlights the need for interdisciplinary and integrative ways of working, which goes beyond integrated or multidisciplinary ways of working between disciplines or services; 2. the use of ‘approach to care and support’, as ‘care’ tends to imply that someone needs to do something for someone that they cannot do themselves, while ‘support’ focuses on enabling someone to live life more independently and provide tools, opportunities and skills to attain this; 3. starting with a focus on ‘palliative’, then ‘geriatric’ and lastly ‘rehabilitative’ to stress the need for an integrative approach without reconfirming the misperceptions that palliative care is only applicable at the very end of life, or rehabilitative approaches only for those who can still recover from their illness, which are both common and widespread misconceptions. |
