# Supplementary material for: A European Association for Palliative Care White Paper defining an integrative palliative, geriatric, and rehabilitative approach to care and support for older people living with frailty and their family carers: a 28-country Delphi study and recommendations
Source: eClinicalMedicine. 2025 Aug 12;87:103403. doi: 10.1016/j.eclinm.2025.103403 (PMC12362020; doi:10.1016/j.eclinm.2025.103403)
Supplement: Appendix 5 [file mmc5.docx]

**Appendix 5. Explanatory texts for the 34 key recommendations^a^**

| **Domain 1. Applicability of a palliative, geriatric, and rehabilitative approach for older people living with frailty** |
| --- |
| **Recommendation 1.1:** **An optimal approach for older people living with frailty should be an integrative palliative, geriatric, and rehabilitative approach, combining the principles or approaches developed in all three disciplines, and centred on the person living with frailty.**  **Explanatory text:** Palliative care, geriatrics, and rehabilitation are disciplines with important and relevant expertise for older people living with frailty.^1–7^ Palliative care is “*an approach that improves the quality of life of individuals and their families facing the problems associated with life-limiting illnesses, through the prevention and relief of suffering by means of early identification and impeccable assessment and the treatment of pain and other problems, whether physical, psychosocial and spiritual”*.^8^ Geriatrics has developed as “*a discipline focused on the needs and priorities of older people, and the approach developed and used over the past decades in geriatric medicine is based on a comprehensive assessment of individuals and delivery of multidisciplinary, person-centred interventions that bridge clinical and social care*”.^9^ Rehabilitation has been defined as “*a set of interventions designed to optimize functioning and reduce disability in individuals with health conditions in interaction with their environment*”.^10^ Over the past decades, each of these disciplines had developed separately, but representatives from these disciplines now find themselves in a shared space where they increasingly recognize the importance to collaborate and seek a common ground.^11–16^ For example, geriatric palliative care has been developed as ‘*a field of inter-specialty collaboration unifying competences from geriatric medicine and palliative care to respond to the socio-demographic changes and challenges of older adults with severe and life-limiting conditions’*.^17^ Rehabilitative palliative care has been defined as ‘*aiming to optimize people’s function and well-being and enabling them to live as independently and fully as possible, with choice and autonomy, within the limitations of advancing illness. It is an approach that empowers people to adapt to their new state of being with dignity and provides an active support system to help them anticipate and cope constructively with losses resulting from deteriorating health’*.^6,7,18^  While each discipline has its own accents and unique contributions to improve the wellbeing of older people living with frailty, these disciplines have many convergent principles, values, goals and overlapping components between them.^1,3,5,6,19–21^ Delivering optimal care for older people living with frailty and their family carers requires an active integration of these approaches to provide integrative palliative, geriatric, and rehabilitative care and support that is centred around the person and their family carers, irrespective of where or by whom that care or support is provided.^5,22,23^ As was noted by one of the professional experts in the Delphi study underpinning this work, “*[…] these clusters of categories [referring to the disciplines] are maybe helpful for practitioners and systems of care but for the person the distinctions are arbitrary”.* |
| **Recommendation 1.2:** **The integrative palliative, geriatric, and rehabilitative approach is, in principle, applicable throughout the frailty trajectory, albeit with varying emphasis on the specificities of each of the three disciplines, depending on the needs and preferences of the person across the frailty trajectory.**  **Explanatory text:** People can develop frailty to various degrees or stages.^24–29^ While there is a well-established consensus among practitioners and researchers that geriatrics is important for older people living with frailty regardless of its stage or degree,^2,30^ the applicability of palliative care for older people living with frailty is most clearly recognised for those experiencing severe, advanced, or advancing frailty,^20,31^ while rehabilitation at the end of life or in palliative stages is not yet widely accepted.^6,7,18^  However, the principles of palliative care – with its focus on quality of life – are relevant and important across the frailty trajectory.^32,33^ Palliative care is not terminal care only but defined as an approach to improve quality of life of patients and families who are facing problems associated with life-threatening, advanced or serious illnesses.^8,34^ Further, personalised rehabilitation is relevant and important throughout the whole frailty trajectory up until death to enable people living with frailty to live as fully and independently as possible until they die.^6,7,18^ Hence, the integrative palliative, geriatric, and rehabilitative approach to care and support is applicable from diagnosis of frailty onwards.^2,6,7,18,20,30,35^ Undoubtedly, as frailty advances, complex problems can arise which may necessitate specialized palliative care and support. |
| **Recommendation 1.3:** An integrative palliative, geriatric, and rehabilitative approach for older people living with frailty and their family carers is applicable across all care and support settings and services. This includes (acute and community) hospital care, primary care, outpatient and ambulatory care, hospice care, intermediate care, care at home and care in nursing or care homes (long-term care facilities) and applies also during transitions between settings.  **Explanatory text:** There are large variations in the way care and support for older people living with frailty and their family carers is organized between countries, regions, and healthcare systems; in the length of time people spent in different places of care; and who is involved in that care, professionally or otherwise.^5,35–37^ Support provided by/to family carers might also vary considerably.^38–40^ Irrespective of these variations, this integrative palliative, geriatric and rehabilitative approach is applicable as an underlying approach across and within all contexts.^5,41^ As highlighted by some of the Delphi panellists, this recommendation does not imply that only specialist clinicians should deliver care; but rather it promotes a general integrated approach to care and support of older people living with frailty, equally applicable to generalists and specialists. |

| **Domain 2. Holistic person-centred care and support focused on capacities as well as needs** |
| --- |
| **Recommendation 2.1:** Care and support for older people living with frailty should be person-centred and tailored to the older person’s needs; these can be functional, physical, psychological, spiritual or existential, social, or practical. Needs assessment should be comprehensive, multidimensional, and holistic, and revisited on a regular basis. As many older people living with frailty develop multiple conditions throughout their trajectory, identifying the cumulative effects of multiple co-existing conditions is necessary.  **Explanatory text:** Epidemiological research among older people living with frailty in different care settings has shown a wide range of health and social care needs in this population.^42,43^ Older people living with frailty often have several co-morbid conditions that can have large cumulative effects and profound detrimental impact on their functional status and well-being.^44^ These include cardiovascular diseases such as heart failure, cerebrovascular diseases such as stroke, and in particular neurocognitive degenerative disorders such as dementia.^45,46^ At the same time, quality of life and well-being of older people are not only determined by functional or physical health problems but also by mental health issues, social context, and practical and existential/spiritual needs.^33,47–53^ Hence, to provide optimal care and support to older people living with frailty, it is important to actively and regularly screen or assess their needs across these multiple domains. This should be done in a holistic manner, acknowledging the interconnection between needs. Such assessment can be done with validated assessment or screening tools, such as the evidence-based method of comprehensive geriatric assessment or palliative care needs assessment tools.^54–60^ |
| **Recommendation 2.2: Complementary to a focus on needs, quality of life and well-being should be promoted by focusing on empowerment and enabling intrinsic capacities. This implies to not only consider weaknesses or deficits of older people living with frailty, but also their strengths and assets, and supporting those to the fullest.**  **Explanatory text:** Older people living with frailty are often characterized in a stereotypical way, focusing on the states of vulnerability, accumulated losses, health deficits, illnesses, burden, and needs associated with these states. However, it is equally important to focus on people’s intrinsic capacities or capabilities, their adaptive coping strategies and resources, their resilience and health assets, and how these can help optimize quality of life and well-being.^61^ Alongside multidimensional needs assessment (recommendation 2.1), it is important to proactively identify their intrinsic capacities, and to work with the person to preserve and optimize these intrinsic capacities in subsequent support and care planning.^62–65^ The World health Organisation (WHO) has captured this in their work on intrinsic capacities, a dynamic concept representing all physical and mental capacities that a person can draw on over a lifetime.^61^ Functional ability comprises the health-related attributes that enable people to be and to do what they have reason to. It is made up of the intrinsic capacities of the individual, the relevant environmental characteristics, and the interactions between these two factors.^61^ Whilst a dual needs- and capacity-based approach represents the optimal way forward to enhance the life of older people living with frailty across the entire illness trajectory until death, there is a dearth of studies showing how to best achieve this in practice.^66^ We therefore urgently need to build an evidence base for this. |

| **Domain 3. Goal-oriented and pro-active approach to care and support guided by values, priorities, and preferences** |
| --- |
| **Recommendation 3.1: Care for older people living with frailty should be goal-oriented and based on people’s priorities, personal preferences, and underlying values i.e. on what matters to them. This includes the setting of realistic and attainable person-centred goals and regular re-evaluation of them.**  **Explanatory text:** It is important to recognize that people have different priorities, goals, and preferences or wishes for the way they want to live their life and consequently for the support, care and treatment they hope to receive.^67^ These priorities, goals, and preferences or wishes are both personal and dynamic, possibly changing over time. A recent meta-review on the perspectives of older people (not specific to frailty) identified several domains important for their quality of life, including health perception, autonomy, life roles and activities, relationships, having a positive outlook, feeling at peace, spirituality, and feelings of security and safety.^68^ The relative importance and meaning of these domains depend on one’s circumstances but also on personal preferences, and all these aspects are intertwined and influence each other.  As recognized within the WHO’s Healthy Aging framework, understanding what matters to an older person, including those living with frailty, and enabling them to be and to do what they have reason to value, should be the central goal of care and support.^37^ Person-centred goal setting is a key tenet of rehabilitative palliative care, recommending health care professionals should elicit people’s values, priorities, and preferences, and work in partnership with each person to identify their personal goals. Such an approach is recommended to switch the focus of care from “What is the matter with you?" to “What matters to you?” and to draw more attention to the personal goals of each person, rather than on the professionally-defined diagnosis and goals of care.^69^ Whilst goals should also be as realistic as possible, the process of supporting people to work towards their person-centred goals has been found to be more important than actually achieving them.^70,71^ The effort to work towards person-centred goals represents an empowering approach that enables older people living with frailty to explore and to come to terms with what is and is not manageable. Although many disciplines have advocated for a shift towards goal-oriented care for older people living with frailty, there is a paucity of evidence on how it can be implemented in practice, or which tools are best to help professionals provide goal-oriented care.^72^ |
| **Recommendation 3.2: Planning of care and support for older people living with frailty should be pro-active instead of reactive alone, and this throughout the whole trajectory. Although the timing of decline or death is difficult to predict, indicators of deterioration or change should be recognized in a timely manner.**  **Explanatory text:** Individual prediction of when people living with frailty will die has been proven difficult.^73–75^ The surprise question ‘would you be surprised if your patient dies in the next 6 to 12 months?’ has been found to have poor to moderate sensitivity/specificity in frailty.^76–79^ Multidimensional care needs in older people living with frailty are frequently unpredictable; they may increase either incrementally or suddenly during acute events or crises. Whilst appropriate reactive responses are warranted, different scenarios can and should also be proactively considered and planned for (see also recommendation 3.4).^80–82^ It is important to recognize that the disease trajectory is progressive for most older people, and frailty is a significant predictor of death.^44,83^ A cycle of reactive care provision could sometimes lead to inappropriate life-saving interventions such as inappropriate hospitalizations at the end of life that may further exacerbate older people’s needs.^80–82^ Preventing and interrupting a cycle of reactive care provision is therefore important to plan care and support pro-actively together with the older person and their family. As estimated life-expectancy does not provide a sufficient basis to guide such pro-active care planning, it is imperative to timely recognise possible indicators of deterioration. Tools to help identify people who might be at risk of deterioration usually combine disease-specific criteria with clinical indicators of decline (e.g. SPICT or Think Ahead tools).^84^ Ongoing research is investigating whether clinical tipping points indicative of increasing needs can be identified in the frailty disease trajectory.^85^ Clinical tipping points are moments at which a series of small changes in a complex system leads to a shift that permanently and substantially alters that system.^86,87^ |
| **Recommendation 3.3: Older people living with frailty should be offered the opportunity to discuss advance care planning (ACP). ACP practices should follow up-to-date evidence-based guidelines and recommendations. These highlight the importance of an ongoing process of reflection and communication focused on values, goals and preferences for future treatment and care, and the involvement of family (if present), next to health care providers. While a sole focus on advance directives should be avoided, ACP encourages people to identify a personal representative and to record and regularly review their preferences.**  **Explanatory text:** Advance care planning (ACP) has been defined in different ways for several populations over the past decades.^80–82,88–91^ Common elements in these definitions include ACP being a continuous process of reflection and communication about personal values, life and care goals, and preferences for future care/treatment, and involving healthcare providers and family (as desired). Outcomes of this process can be recorded (e.g. in advanced directives) and need regular reviewal. Recent work emphasizes the need for a broad holistic approach to ACP, which is not only focused on medical aspects concerning the end of life, but also on what matters to people in their daily lives, their preferred activities and what gives their life meaning.^88–90^ As such, it overlaps considerably with other concepts, such as goal-oriented and person-centred care.^69^  Although widely advocated for people with serious illnesses, including those living with frailty, there have been recent debates around ACP including concerns about its effectiveness and implementation.^92–98^ Overall, most researchers and practitioners involved in the care for older people highlight the importance of offering people the opportunity to discuss ACP and not reduce it to filling in advance directives, as tick box exercises have a little chance of influencing the quality of end-of-life care and dying.^91,99–103^ Messaging around ACP should set realistic expectations about the benefits of ACP, as not all end-of-life situations can be foreseen, planned, or controlled.^104^ As evidence of ACP continues to build through ongoing research, it is important for professionals to follow up-to-date and evidence-based guidelines and recommendations, following existing legal frameworks appropriately.^105^ Crucial to conducting ACP is preventing pitfalls (e.g. documentation without communication) while optimizing its benefits (e.g. offering people an opportunity to reflect, discuss and consider what is important to them and their future care).^5,106–109^ |

| **Recommendation 3.4: It is important to acknowledge clinical uncertainty as an inherent feature of a frailty trajectory and engage in parallel planning of care. Parallel care planning means to consider a range of potential outcome options for care (e.g. simultaneous parallel planning for recovery and deterioration in health), to offer to discuss this with patients (and family carers, if present), and plan care in parallel with them.**  **Explanatory text:** The prognostic uncertainties and unpredictability inherent in a frailty illness trajectory can serve as barriers to engage in pro-active care planning^31,110,111^. Although not everything can be planned, offering to engage in parallel care planning together with the person and family carers (if available and desired) could be a way to consider and discuss a range of potential outcome options for care.^18,112–114^ Parallel care planning considers a range of potential outcomes and options for care (e.g. simultaneous parallel planning for recovery and deterioration in health), offers to discuss the outcomes and options with patients (and family carers, if available), and plans care in parallel with them. Over time, some scenarios become more obvious, while others will become less likely. Both recovery and death would be possible outcomes and clinicians should work with patients and families with this understanding in mind.^18,72,114^ |
| --- |
| **Recommendation 3.5: Emergency response and out-of-hours planning (i.e. what to do in case of an emergency or out of hours) should be an important part of a pro-active care plan to support older people living with frailty, their family carers, and involved professionals.**  **Explanatory text:** Emergency response planning is an inherent feature of pro-active care planning by preparing people to respond appropriately to emergencies.^115,116^ An emergency response plan could be a documented series of steps or actions that could be taken during a critical event, exacerbation, or emergency, which would ensure optimal care can be delivered corresponding to people’s needs and preferences. Communication should be done in a sensitive and person-centred way. If done well, it could help people access the care they need and at the same time prevent them from receiving inappropriate or futile care/treatments. Out-of-hours planning (i.e. who to reach and what to do when services need to be reached after regular office hours) seems to be particularly essential in this population.^117,118^ In many countries, access to appropriate care and support is difficult out-of-hours, although the organization of care varies substantially between and even within countries.^119^ Despite the need for high-quality emergency response and out-of-hours planning, research into effective interventions within this topic is lacking. Out-of-hours palliative care has been identified as an important research priority, also by patients and family carers themselves.^120–123^ |
| **Recommendation 3.6: Care for older persons living with frailty can include interventions such as medication review to ensure people are only taking medications that are likely to be beneficial for them. Such interventions aim to support people to remain well and independent for as long as possible and reduce the risk of ill health and poor outcomes.**  **Explanatory texts:** Research has shown that older people living with frailty often take many medications, some of which are likely not beneficial for them.^124,125^ Regular and sensitive communication and decision-making regarding medications is important throughout the entire frailty illness trajectory. A recent overview of systematic reviews concluded that deprescribing interventions in older adults are likely to result in medication reduction but evidence on other outcomes (i.e. relating to adverse events) or evidence for vulnerable subgroups or settings (e.g. people with dementia, frailty, or multimorbidity) is limited.^45,126^ This review emphasized the need for more large-scale high-quality research, focused on patient-reported outcomes, harms, and effects of deprescribing in specific subgroups. |

| **Domain 4. Communication and shared decision-making** |
| --- |
| **Recommendation 4.1: Person-centred communication and shared decision-making are crucial components of care for older people living with frailty and their family carers. This includes sensitive, open, empathetic, and tailored information and communication, which invites people to participate in shared decision-making, while avoiding stereotyping, prejudicial or discriminatory language.**  **Explanatory text:** Person-centred communication is at the core in most of the recommendations included in this integrative palliative, geriatric, and rehabilitative approach to care and support for older people living with frailty and their family carers. Good communication is a cornerstone of person-centred care, underpinning the quality of care, enhancing relationships between patients, families, and professionals, and is associated with better quality of life.^69,127,128^ The timing, content, style, pace, and setting of communication, can all have a major impact on how people experience care and support throughout their trajectory. Good communication is sensitive, empathetic, open, and tailored to the person’s preferences. Such personalized communication is of particular relevance for older people living with frailty, considering that support and care planning can be highly complex.^6,18,114,129^ Research has highlighted the importance of being sensitive to a diversity of preferences amongst older adults, and to avoid stereotyping or jargon. Although there are variations in information preferences, older adults often like the opportunity to discuss their end-of-life needs and value open, frank conversations. However, they may not initiate these conversations and instead wait for health professionals to raise the topic of end-of-life discussions.^130^  Shared decision-making has been defined as ‘*a process in which decisions are made in a collaborative way, where trustworthy information is provided in accessible formats about a set of options, typically in situations where the concerns, personal circumstances, and contexts of patients and their families play a major role in decisions’*.^131^ It should facilitate discussions between professionals and older persons when decisions must be made about desired care and treatment. Although poor health and the clinical complexities and uncertainties can pose major barriers to participate in shared decision-making, an explicit invitation is important to older adults.^132,133^ However, with some exceptions, shared decision-making models with specific focus on older people with living frailty are limited, and better evidence is needed to understand how to implement it in practice.^134^ A realist synthesis indicated key aspects in shared decision-making for older people with multiple health and social care needs, which include face-to-face interactions, permission and space to discuss options, and continuity in the patient-professional relationships.^135^ However, for it to work in practice, a radical shift is needed from a biomedical focus to a more person-centred ethos. Service providers need to be open to change their professional behaviour and reorganise their services in practice. |
| **Recommendation 4.2: As capacity is decision-, time-, and context-specific, it is important to tailor communication to the cognitive and decisional capacities of the person living with frailty, and to support the person as much as possible, by using easy-to-read-and-understand materials or creating adequate contexts.**  **Explanatory text:** Capacity has been defined as ‘*the ability to use and understand information to make a decision, and communicate any decision made*’.^136^ From a legal perspective, people often either do or do not possess capacity. However, clinically, decisional capacity is a state that can be lost and regained; it is issue- or task-specific and can fluctuate over time and depend on context.^137^ Therefore, it is important to always start with the assumption that a person has capacity, tailor communication to the person’s capacities, and be alert for opportunities to discuss current and future care preferences with people who have declining capacity. Supporting people with declining capacity in conversations seems to be an important ethical imperative. Providing information in adapted formats (e.g. verbal, visual, in writing, using examples) and creating optimal contexts for sensitive communications (e.g. distraction-free environments, hearing aids) are examples of ways to support and empower people.^129,138–141^ This is important for people who develop dementia or other diseases that could result in diminishing capacity over time. As highlighted in Boyle’s research, people with dementia, including those with advanced dementia, should be regarded as being able to exercise agency.^142^ Although they might lack deliberate decision-making capacity, they can nonetheless demonstrate creative capacity for agency.^143^ |

| **Domain 5. High-quality end-of-life care, and dying with comfort and dignity** |
| --- |
| **Recommendation 5.1: It is important to recognize when an older person with frailty may be dying, to understand the process of dying, to sensitively communicate with patients and families about dying (tailored to their information preferences), and to provide care for dying persons using best-practice guidelines.**  **Explanatory text:** While some people living with frailty might die suddenly, for most of them, dying is a slower and gradual process. Importantly, there are signs and symptoms common to the dying process.^73,74^ Recognizing and monitoring these patterns is hugely important for clinical decision-making. Medications for terminal restlessness, pain and excessive secretions can be anticipated and appropriately prescribed to prevent distress and promote comfort in the last days of life.^144^ Several checklists or pathways have been developed over the past decades to help clinical staff recognize the terminal phase of life and help guide care planning for the last days of life.^145,146^ While some programs have shown successful implementation and outcomes,^147^ others have been abandoned due to unsafe and unintended usage (such as the Liverpool Care Pathway).^148^ It is important to recognize that any tool or document that aims to standardize care practice requires human expertise to work. It is people who work with tools and the underlying values and culture in which a tool is embedded is a crucial component of their success. Therefore, last-days-of-life checklists or pathways may help to guide care but only if implemented as part of a high-quality palliative care culture, with attention to training of people working with them. Finally, sensitive communication with patients themselves as well as their families is a crucial part of good terminal care. Communication with families about what to expect during the final days of life can help them feel less frightened or uncertain.^149,150^ |
| **Recommendation 5.2:** **As the death of many older people who live with frailty often is preceded by one or several medical end-of-life decisions, evidence-based and careful decision-making and communication about such decisions with patients and their families is needed.**  **Explanatory text:** Research has shown that the dying process of older people is often preceded by medical decisions concerning the initiation, continuation, or discontinuation of possibly life-prolonging treatments, such as decisions about (artificial) feeding or hydration, or the intensification of pain and symptom management.^151,152^ Such decisions require careful consideration and communication, with patients and usually also with family carers.^153,154^ In general, any end-of-life decision should follow up-to-date evidence-based clinical and ethical guidelines, such as those produced by Cochrane, NICE or national Evidence-based Medicine Centres.^146,155,156^ Moreover, as end-of-life practices are not only embedded in a medical and ethical context but also in a legal and regulatory, it is important for clinicians to know and understand these contexts and to operate within their boundaries. |
| **Recommendation 5.3:** **To enable dying well among older people living with frailty, it is important to communicate sensitively with people about preferences and possibilities on where and how they would prefer to live and die (taking into account their information preferences), what a dignified end of life means to them, and enable them, as much as possible, to die following their preference and choice.**  **Explanatory text:** What it means to die well is highly personal and socio-culturally embedded.^157^ Communicating in a sensitive manner and considering diversity in preferences and values is a crucial precondition to die well.^158^ While there is a lack of globally validated frameworks articulating what entails a good or dignified end of life or death, from a person-centred point of view, it is important to start from the reference framework of the person him/herself and to work from there to ensure a good death. This can include preferences regarding health, care and life in general (including living arrangements, social or everyday activities).^159^ Importantly, preferences and choice-making processes are often dynamic, relationally entangled and evolve over time.^104^ This underscores the need for ongoing communication with patients and family carers, and for revisiting preferences.^108^ Furthermore, decisions about care and support will always need to be made within the boundaries of what is realistically possible. For example, while many older people prefer to die at home, a nursing home admission at the end of life is not always preventable. Hence, open and honest conversations about possibilities and limitations are important. |

| **Domain 6. Family carers (if available) as provider and recipient of care and support** |
| --- |
| **Recommendation 6.1:** **Family carers of older people living with frailty can be family members, close friends, befriended professionals, neighbours or others who are in a close supportive or caring role, have strong emotional and social bonds with, and share in the illness experience of the person living with frailty. If family carers are available, they play a crucial role in providing care, rehabilitation, and tangible support to older people living with frailty, in different places of care. Therefore, they should be considered as potential partners in the care planning and decision-making process with whom to build joint partnerships, as preferred by the older person and themselves.**  **Explanatory text:** Although much literature focuses on professional caregiving, considerable support and care for older people living with frailty is provided by family carers. Family carers are not only people who have a blood relationship. They can be close friends, befriended professionals, neighbours or others. Support provided by family carers can be physical, practical but also emotional, social or spiritual. Research has shown that family carers are essential for people with frailty and their support plays a crucial role in many outcomes relevant to older people, including enabling older people to stay at home at the end of life.^38–40^ Family carers should be recognized for their crucial role and should be engaged as equal partners in care and support in the whole planning and decision-making process, as deemed appropriate by the older persons themselves.^153^  In both clinical practice and research, further work is needed to understand how family carers can be optimally engaged in the co-support and care of older people living with frailty, including how to deal with difficult relationship dynamics. New models to achieve this are being developed but need further study. For example, in Belgium, the ‘Trialoog’ program aims to improve the relationship and trust between resident, their family carers, and the nursing home.^160^ It is based on relationship-focused and participatory principles and explicitly aims to move beyond mere needs-based principles. It focuses on jointly and actively building a good everyday life in the nursing home. |
| **Recommendation 6.2:** **Next to providing care, family carers should be recognized as potential care recipients as they might at times become overburdened and in need of support themselves. Respite care, tailored support, training or education for family carers should be focused on their needs as well as on empowering their intrinsic capacities and supporting their adaptive coping strategies.**  **Explanatory text:** Family carers might need support or care themselves at times.^161^ Research found that carers’ needs differ from those of patients and therefore should be assessed separately, through a personalized holistic assessment.^162–165^ Needs have been identified in two domains: 1) Needs related to being ‘care partners’ in supporting patients. This includes needs for information, skills or equipment to fulfil this role; and 2) Needs related to being ‘clients’ in their own right to preserve their own well-being and health (in the broadest sense including physical, emotional, practical, social and existential), their identities, and personhood.^162,163^  As individual family carers differ in what they need and can do, it is important to regularly assess and monitor needs and tailor support accordingly. One of the most comprehensive evidence-based tools available is the Carer Support Needs Assessment Tool or CSNAT.^163,166^ The tool is delivered as part of a person-centred process of assessment and support, enabling carers to identify, express, and prioritise domains where they might need more support, followed by a needs-led conversation to enable the co-creation of a personalised support plan.  Achieving comprehensive, person-centred carer support at the end of life warrants a whole system change and cultural shift, both at practitioners and organizational levels. This was evident from the 2018 report from Hospice UK bringing together research evidence and practice experiences from stakeholders providing recommendations at all levels.^162^ |
| **Recommendation 6.3:** **There should be attention to anticipatory grief and after-death bereavement support for family carers, including involving community support initiatives and specialist mental health support if needed.**  **Explanatory text:** Supporting family carers should not stop when a person dies, as it should include support after death into bereavement. This became apparent in the Covid pandemic that disproportionally affected older people with frailty or comorbidities.^167^ Grief is an emotional process when losing someone important; mourning is an expression of that grief and is highly culturally and historically embedded. Bereavement is the experience of having lost someone important. Grief and bereavement are natural parts of life and, for most people, not pathological. Support from the bereaved person’s network and community, including friends, families, and neighbours can be very important for people. Initiatives such as the Good Grief Festival aiming to make grief a less lonely experience could enhance their social capacity. In many countries, bereavement support services, including those provided by funeral services or spiritual/existential support, are available; although sometimes not well known to the public.^167^ In some cases, specialist mental health support is recommended.^168^ An estimated 10% of bereaved people are diagnosed with ‘prolonged grief disorder’.^169^ Overall, a tiered stepped approach based on needs has been advocated.^170^  In the context of dementia and other neurodegenerative diseases that might co-exist with frailty, anticipatory grief, that is, grieving before a death has occurred, is a well-described phenomenon. A person with dementia may be ‘lost’ to the family due to personality and relationship changes.^45^ Also, as people move towards residential care setting, physical absence can enhance this feeling, further inducing feelings of guilt and a sense of failure, particularly in spouses.^171^ In addition, while access to more specialised support for complex grief may be needed at times, access to local services and community initiatives represents adequate support for most people. |

| **Domain 7. Integrated, interdisciplinary care and support, and access to services** |
| --- |
| **Recommendation 7.1:** **An integrated care approach with attention to ensuring coordination and continuity of care for older people living with frailty and their family carers is needed. This should be centred around the needs of older persons, their families, and their communities. Particular attention is needed to integrate health care, social care, community and voluntary support, and to optimizing communication and collaboration when people move between settings or carers. Case managers or care coordinators could support this process.**  **Explanatory text:** Integrated care is a priority formulated within most health systems, and specifically for people living with multiple chronic diseases such as people with frailty.^15,172^ Older people living with frailty often have several care providers and regularly experience transitions between care settings, particularly when closer to death.^172–177^ Integrated care has been described as the opposite of fragmented or episodic care and is used interchangeably with terms such as coordinated, seamless or shared care. Although there are several definitions of integrated care (definitions can be process-based, user-led, or health system-based), a central feature to all is the importance of providing care centred around the needs of individuals, their families, and their communities.^175,178^ Worldwide, different types of integrated care models have been developed.^2,179^ Some are individual models of integrated care (e.g. focusing on case management, or individual care plans), others are group- or diseases-specific models (e.g. chronic care model, or integrated palliative care models for older people living with frailty),^50,179–187^ or population-based models (e.g. Kaiser Permanente US).^188^ Several core components can be identified across the different integrated care models, including enabling patient engagement and self-management support; developing a multi-professional working culture; adopting evidence-based clinical pathways and protocols; aligning incentives; effectively managing resources; continuously monitoring and improving performance; and investing in supporting information technologies. One model is not necessarily superior to another model. Any integrated model is contextually bound and can only be successful if tailored to the wider health care context and needs of the populations it serves.^178^ The large body of literature shows positive effects of many of these models but highlight that evidence on cost-effectiveness often remains controversial. Finally, while there are exceptions, few models of integrated care have been specifically designed for frailty. Case management for integrated care for older people with frailty has been studied but evidence is uncertain.^189,190^ Existing frailty models often do not explicitly mention palliative care. Through this White Paper, we argue the need to integrate palliative care in integrated care models for older people living with frailty. Initiatives in the UK and Belgium have been developed but large-scale implementation is lacking.^180–182^ |
| **Recommendation 7.2:** **Interdisciplinary team-based collaboration (i.e. working collaboratively and using shared guidelines and protocols) is needed, with those working from different professions and sectors delivering services in a collaborative way with the older person with frailty and family carers at the centre. This should result in a personalized and shared care planning process in which goals are set, and decisions are made, collaboratively.**  ﻿  **Explanatory text:** Interdisciplinary team-based collaboration aims to integrate perspectives of team members through interaction and usually integrate different disciplinary approaches into a single consultation. The older person with frailty and their family carer (if present and preferred by the person with frailty) are at the centre of this collaboration.^189^ Interdisciplinary collaboration is preferred over multidisciplinary team approaches in which the expertise and experience of people from different disciplines is used, but each discipline approaches a person from their own perspective. Usually, multidisciplinary team meetings are held in the absence of the patient. Although interdisciplinary models of working would be considered truly person-centred and crossing disciplinary boundaries, evidence of effective and workable models seems limited.^191,192^ This is an important area for future innovation and research. |
| **Recommendation 7.3:** **Timely and needs-based access to health and social care or support services for all older people living with frailty is important, particularly access to rehabilitation, and to social and palliative care services, as referral is often too little and too late. Several models and interventions to increase timely access to services are promising. Next to age, other social and structural intersectional determinants of health impacting access to these services, need consideration.**  **Explanatory text:** Having timely access to care and support is a central component of our integrative approach in frailty, with timely implying ‘when needed’. Access is closely related to availability of services and the characteristics of the health care system, hence it depends on, among other factors, where people live. While access is a generic issue, the specific lack of timely access to rehabilitation, social community services and palliative care services for people living with frailty has been cited as a priority.^30,193^ Although this White Paper underlines the importance of a joint collaborative approach across disciplines, it is important to ensure referral to specialist services of any kind when needed, such as when needs become complex, atypical, or require specialist care or advice.  Interventions specifically aimed to improve access are navigation programs, using paid or volunteer navigators (sometimes called linked or community workers, social prescribers, or other).^194–197^ A recent evidence-informed policy brief from the WHO Regional Office for Europe on navigation concluded that, although evidence of their effectiveness is still limited, existing studies do indicate that navigators can contribute to improving access and continuity of care and are a promising approach to better integration of care, particularly for disadvantaged populations.^198^ As many intersecting social and structural determinants of health impact the services and resources people are able to access, an intersectionality-based approach to ensuring timely and needs-based access seems a crucial consideration for future approaches. |

| **Domain 8. Care and support by competent professionals** |
| --- |
| **Recommendation 8.1:** **Professionals who care for and support older people living with frailty and their family carers should develop generalist palliative, geriatric and rehabilitative care competencies (knowledge, skills and attitudes) through under- and postgraduate education and training, as part of a life-long learning trajectory.****Explanatory text:** As most health and social care professionals encounter older people, including those with frailty and their family carers, developing competencies that are in line with the proposed integrative approach is crucial. Competencies include knowledge (understanding of the concept), skills (specific abilities to show behaviour), but also attitudes and values (internal drivers of behaviour) underlying this approach.^199,200^ As pointed out by some of the older person’s representative panellists in the Delphi study underlying this work, values such as empathy, respect, and kindness, represent key determinants of how professionals engage with older people. Interdisciplinary and interprofessional collaboration, particularly when complex situations arise, should also be a key component in education. Developing such value-based competencies should be part of standard curricula and of a life-long learning trajectory. As professionals working with older people living with frailty critically reflect on their practice, this framework could serve as a standard to both benchmark and guide improvement efforts in their provision of care and support. |
| **Recommendation 8.2:** **In all places where older people with frailty live, continuous quality improvement initiatives should be set up to ensure high-quality evidence-based care and support are provided.** **Explanatory text:** Continuous quality improvement can be defined as a progressive incremental improvement of processes, safety and patient care.^201^ As patients, systems, and societies are constantly evolving, incorporating some way of continuously monitoring and reflecting on how care or support is provided, is an important aim for care providers, organisations, communities, and governments involved in caring for and supporting older people living with frailty. Common methodologies used in quality improvement initiatives include the Plan-Do-Check-Act (PDCA) cycles or other tools to help structure this process.^202^ Unique initiatives in long-term care include the creation of Living Labs. For example, the Maastricht’s Living Lab in Ageing and Long-term care has developed a sustainable model for translational and interdisciplinary research to improve quality of life, care and work, by bridging research, practice, education, and policy, ensuring the best evidence is used in everyday care practice and research seeks to address the needs surfacing in practice.^203^ Older people and their relatives and representatives also play a central role in these models, as patient and public engagement, involvement and participation is an important aspect of future research, practice, and policy.  At the policy level, interesting developments around quality regulation in long-term care include the integration of narrative methods alongside the more traditional use of quality indicators, as is currently being studied in the Netherlands.^204,205^ |
| **Recommendation 8.3:** **The workforce of health and social care professionals who provide care for and support older people living and dying with frailty and their families should be resilient, qualified, and respected, and support for them should be available as needed.** **Explanatory text:** Increasing labour shortage is currently being experienced by the long-term care sector in Europe and beyond. An increasing proportion of professionals is older than 50 years old. Considerable challenges have been identified in the long-term care sector around working conditions (i.e. physical, social and psychological challenges).^206^ Hence, support for the workforce providing care for people living with frailty should be prioritised. A recent Organization for Economic Cooperation and Development (OECD) report called for a comprehensive policy strategy to tackle poor working conditions and insufficient social recognition of long-term care work; attract workers in the sector; and avoid labour shortages reaching unacceptable levels.^207^ Such a strategy should cover several dimensions, with different priorities across countries depending on their specific context. It can include raising of wages and staff requirements, increasing public financing, supporting social dialogue, strengthening training, increasing use of new technologies, and strengthening health prevention policies. The integration of palliative care approaches in the long-term care sector could positively influence mental and physical health and well-being of employees. For example, the integration of compassionate workplace values alongside education and support for employees when confronted with end-of-life experiences could be highly beneficial, although evidence for such approaches is lacking. |

| **Domain 9. Contextualized and culture**-**congruent care and support** |
| --- |
| **Recommendation 9.1:** **An integrative palliative, geriatric and rehabilitative approach should be delivered in any type of health care system or setting, specialist or non-specialist, and in various health care systems and contexts.**  **Explanatory text:** The integrative approach outlined in this White Paper with all key domains and recommendations is applicable in all health care systems and places where people live and die. Of course, there will be different priorities across countries, systems or settings, depending on the specific context. Differences in legal and regulatory frameworks, geography and epidemiology, socio-cultural and socio-economic backgrounds will influence priorities and possibilities. However, the underlying values and principles behind this integrative approach should be applicable anywhere. One important limitation to our work is that literature and the involved experts are not globally representative. Hence, future work should focus on studying and integrating the views of stakeholders from all parts of the world to further refine and validate the approach. |
| **Recommendation 9.2:** **Culturally competent and culturally congruent care provision is important for all older people living with frailty and their families, regardless of their age, gender, social situation, background, ethnicity, language, religion, spirituality, nationality, origin or other characteristics. It implies professionals strive to work within the cultural contexts of the persons they care for, a process which involves the integration of cultural awareness, knowledge, skills, and sensitivity.**  **Explanatory text:** Many societies are not only aging but are also increasingly becoming more diverse.^208–212^ It has been argued that care is not always aligned with people’s different backgrounds, and in some cases, this goes as far as racism and discrimination. This recommendation explicitly aims to acknowledge and reject such practices and underlines that care and support should be provided with respect to people’s personal values, beliefs, and world views, regardless of their demographic, socio-cultural, or other backgrounds. Future individuals, organisations, communities, policymakers, and societies should become aware of the need for culturally competent and culturally congruent care and take action to improve it. While it is still unclear how to effectively do this, it will be important for all actors to recognize the role they can play.^213^ |
| **Recommendation 9.3:** **An integrative palliative, geriatric and rehabilitative approach should always be provided with respect for patient and human rights.**  **Explanatory text:** In line with recommendation 9.2, a human rights-based approach means that all forms of discrimination in the realization of rights must be prohibited, prevented, and eliminated. The Universal Declaration of Human Rights (1948) was a milestone document in the history of human rights.^214^ It set out, for the first time, fundamental human rights to be universally protected. It paved the way for the adoption of more than seventy human rights treaties, applied today on a permanent basis at global and regional levels. The United Nations Principles for Older Persons were adopted in 1991, incorporating 18 principles that are grouped under five themes: independence, participation, care, self-fulfilment and dignity.^215^ Within healthcare, patient rights and capacity legislations have been adopted in many continents and countries, aimed to protect the rights of every patient in terms of quality care, information and consent, freedom of choice, protection of privacy, and other. These are very country- or context-dependent. Specifically, palliative care has been recognized explicitly by the WHO as part of the human right to health, and in some countries, laws exist on palliative care as a basic human right.^216,217^ |

| **Domain 10. Community and public health approaches** |
| --- |
| **Recommendation 10.1:** **Community initiatives and public health approaches should be promoted, including compassionate, caring, or healthy aging communities or neighbourhoods embracing aging and dying as normal social parts of life.**  **Explanatory text:** Although the fields of aging and palliative care are relatively independent, both have evolved towards emphasising the need to recognize the impact of all levels of the socio-ecological model on the health and well-being of people. In both fields, the importance of a public health approach is stressed, implying the need to recognize the impact of policies and communities beyond the clinical or health care context.^41,218,219^ In the field of aging, this has led to the development of age-friendly communities. In the field of palliative care, compassionate communities and public health approaches have developed. The creation of supportive environments and community actions are seen as an important way forward to embracing aging and dying as normal social parts of life. This also implies the need to position aging and dying not only from a needs-based perspective but also from an asset- or capacity-based perspective.^37,220^ While community development in this direction is apparent, more evaluation research is needed to fully understand their impact and mechanisms.^221–223^ Moreover, it is important for the age-friendly and compassionate communities to collaborate, as currently, these initiatives, although complementary in their objectives, have not yet converged in practice.^221^ |
| **Recommendation 10.2:** **Communities and volunteers play an important role in supporting older people living with frailty, as do families and professionals. Community members and volunteers should receive adequate resources, support, tailored training, and respect.**  **Explanatory text:** The importance of volunteers is well recognized, particularly in the field of palliative care.^224–230^ Volunteering concerns an act in which time is freely given, with no expectation of financial gain, and can be done in the context of palliative care. Volunteering can be situated within grassroots, bottom-up structures (e.g. community initiatives), or top-down formal structures e.g. coordinated within a healthcare service). Volunteers cannot replace qualified staff and need to be regarded as complementary.^229^  Studies have shown the impact volunteers have at the end of life, offering practical, emotional, informational, spiritual, and bereavement support; providing a link between health care services and the community; and increasing social capital and cohesion.^230^ Several reviews in the field of older persons’ acute care, community-dwelling seniors, and residential aged care have also concluded the valuable role of volunteers to improve health outcomes for older adults including those with frailty.^195,224,231–235^  Importantly, as supporting people living with frailty and their families can be challenging, there is a need for adequate resources and support for the volunteers themselves when implementing volunteer programs.^226,231^ This can include tailored training, coaching, and role definition, and strategies to support the volunteers to feel valued and appreciated. Tailoring is important as different settings might have different requirements and volunteers can work across the continuum of care and support for older people with frailty. Volunteer programs can have benefits for their recipients and for the volunteer themselves as research among older adults’ volunteers has shown.^231^ |
| **Recommendation 10.3:** **Older people living with frailty, and their family carers, should be valued in society throughout their life, and social exclusion should be prevented.**  **Explanatory text:** Older people are too often marginalized and are perceived as a burden, particularly those with ongoing losses in capacities and independence.^220,236^ In addition, it has been described extensively how pervasive and complex social exclusion in later life manifests itself as a societal issue in a globalized world. It can implicate interconnected economic, social, service, civic (civic participation and socio-cultural), and community and spatial domains of daily life. ^237–239^ The high risk of social exclusion applies to all older people, and particularly to those living with frailty, as disabilities, comorbidities and functional or cognitive decline might even worsen their situation. Older persons, including those living with frailty, should be valued in life, for being humans. Several of our Delphi panellists stressed the importance of seeing and valuing older people beyond their social or economic contributions. Older people, regardless of whether they live with frailty, should be valued for being humans with considerable life experiences, without any judgment on the value of their previous contribution.^237–239^ |

| **Domain 11. Ethical principles and frameworks** |
| --- |
| **Recommendation 11.1:** **It is important to reject stereotyping, stigmatisation, and discrimination based on age or disabilities as they lead to increased vulnerability and exclusion. These should be prevented via raising awareness, age-appropriate language and policies, and supporting initiatives that make older people visible in society in a realistic way, including those who are frail and need support.**  **Explanatory text:** Stigmatisation based on age (ageism), mental health conditions (mentalism), and disability (ableism) has been well described in the literature.^240,241^ Such -isms refer to the stereotypes (how we think), prejudice (how we feel) and discrimination (how we act) towards others or oneself based on age, mental status or disability.^242,243^ Ageism, ableism, and mentalism also intersect and exacerbate other forms of disadvantage including those related to sex or race.^240,241^ Ageism has been found to be associated with earlier death, poorer physical and mental health, lower quality of life, and slower recovery from disability in older people.^243^ It contributes to long-standing misconceptions and myths that aging societies are not fiscally sustainable, and that older people receive disproportionate benefits at the expense of younger generations.^220,244^ It can be very pervasive for those living with frailty.^245,246^ However, research from the European Observatory’s Economics of Health and Active Ageing series has clearly found that population ageing is not a major problem for the sustainability of health care systems or societies but instead shows ‘win-win’ politics that produce good outcomes for people of all ages by focusing on life-course policies in policy design.^244,247^ Strategies advocated by the WHO to combat ageism are found in policy and law, educational activities, and intergenerational interventions. |
| **Recommendation 11.2:** **All end-of-life decision-making should put the person living with frailty at the centre, preventing over- as well as under-treatment, and ensuring appropriate and proportionate care, treatment, and support, in accordance with preferences and values.**  **Explanatory text:** End-of-life decision-making is a widely debated part of end-of-life care literature, in particular end-of-life decisions with a possible or certain life-shortening effect such as non-treatment decisions, intensified symptom management, euthanasia and assisted dying. Different countries also have diverging laws regulating some of these decisions.^217,248,249^ Regardless of the specific legal and regulatory context, a central value to end-of-life decision-making stipulated in this recommendation is the importance that clinicians who are involved in end-of-life decision-making commit to centre the values of older people living with frailty, ensuring appropriate and proportionate care, treatment, and support, which accords with their expressed preferences and values (see also recommendations under domains 2 and 3). This recommendation is also closely linked to our recommendation 11.1, as stigmatization based on age might also lead to limiting people’s choices in health care. |
| **Recommendation 11.3:** **In times of disruptive events such as disaster, pandemic or war, care and support should remain based on ethical principles and frameworks. Therefore, regular monitoring, reflection, and adaptations of practice or policy are needed before, during and after such crisis periods.**  **Explanatory text:** During the COVID-19 pandemic, healthcare systems were overwhelmed and there were problems with ensuring adequate capacity for healthcare resources, including beds and ventilators.^250^ Age became an intensely debated criterion with regard to allocating scarce resources.^251,252^ However, as many authors have argued, age or the presence of absence of frailty should never be used as a unique criterion for withholding or not initiating life-saving procedures, even in pandemics or cases in which healthcare resources are extremely scarce.^253–256^ Ageism and frailism should be prevented. This is based on fundamental Codes of Ethics that all professionals caring for patients should obey, and on ethical principles of justice, non-discrimination, and human rights. During disruptive events or public health emergencies, governments, organisations, and practitioners should ensure non-discriminatory policies when it comes to medical treatment and care. |

**^a^** Using the same rapid literature review methodology that was explained in Appendix 1, we conducted an additional rapid literature review between September 2024 and February 2025 to identify new empirical evidence to complement the earlier cycles of rapid reviews. These additional rapid reviews further strengthened the explanatory texts for the key recommendations.

**References for the explanatory texts**

1. Pialoux T, Goyard J, Hermet R. When frailty should mean palliative care. J Nurs Educ Pract. 2013;3(7):75–84.

2. Evans CJ, Ison L, Ellis-Smith C, Nicholson C, Costa A, Oluyase AO, et al. Service Delivery Models to Maximize Quality of Life for Older People at the End of Life: A Rapid Review. Milbank Quarterly. 2019;97(1):113–75.

3. Turner G, Clegg A. Best practice guidelines for the management of frailty: A British Geriatrics Society, Age UK and Royal College of General Practitioners report. Age Ageing. 2014;43(6):744–7.

4. Hall S, Petkova H, Tsouros AD, Costantini M, Higginson IJ. Palliative care for older people: better practices. World Health Organization; 2011.

5. Van Den Noortgate NJ, Van Den Block L. End-of-life care for older people: the way forward. Age Ageing. 2022;51(7):afac078.

6. Tiberini R, Turner K, Talbot-Rice H. Rehabilitation in palliative care. In: Duncan Macleod R, Van den Block L, editors. Textbook of Palliative Care. Cham: Springer Nature Link; 2019.

7. Tiberini R, Talbot-Rice H, Turner K. Rehabilitation in Palliative Care: Principles in the Context of Life-Limiting Illness, Practices for the Interdisciplinary Team, and the Specialist Contribution of Physiotherapy and Occupational Therapy . In: Duncan Macleod R, Van den Block L, editors. Textbook of Palliative Care. 2nd ed. Cham: Springer Link; 2025. p. 605–39.

8. World Health Organization. 2021. [cited 2020 Jun 10]. World Health Organization’s definition of palliative care. Available from: http://www.who.int/cancer/palliative/definition/en/

9. Cesari M, Amuthavalli Thiyagarajan J, Cherubini A, Acanfora MA, Assantachai P, Barbagallo M, et al. Defining the role and reach of a geriatrician. Lancet Healthy Longev. 2024;5(11):100644.

10. World Health Organization. Rehabilitation [Internet]. 2024 [cited 2025 Jul 2]. Available from: https://www.who.int/news-room/fact-sheets/detail/rehabilitation

11. Timm H, Thuesen J, Clark D. Rehabilitation and palliative care: Histories, dialectics and challenges. Wellcome Open Res. 2021;6:171.

12. Crocker T, Forster A, Young J, Brown L, Ozer S, Smith J, et al. Physical rehabilitation for older people in long term care. Cochrane Database Syst Rev. 2013;28(2):CD004294.

13. Visser R, Borgstrom E, Holti R. The Overlap Between Geriatric Medicine and Palliative Care: A Scoping Literature Review. Journal of Applied Gerontology. 2021;40(4):355–64.

14. Pacala JT. Is Palliative Care the “New” Geriatrics? Wrong Question — We’re Better Together. Journal of the American Geriatric Society. 2014;62(10):1968–70.

15. World Health Organization. Integrated care for older people (ICOPE): Guidance for person-centred assessment and pathways in primary care, second edition. Geneva; 2024.

16. Coker JF, Martin ME, Simpson RM, Lafortune L. Frailty: An in-depth qualitative study exploring the views of community care staff. BMC Geriatr. 2019;19(1):1–12.

17. Voumard R, Rubli Truchard E, Benaroyo L, Borasio GD, Büla C, Jox RJ. Geriatric palliative care: A view of its concept, challenges and strategies. BMC Geriatr. 2018;18(1):220.

18. Tiberini R, Richardson H. Social Care Online from SCIE. 2015 [cited 2025 Mar 25]. p. 65 Rehabilitative palliative care: enabling people to live fully until they die: a challenge for the 21st century. Available from: https://www.hospiceuk.org/publications-and-resources/rehabilitative-palliative-care-enabling-people-live-fully-until-they-die

19. Hall S, Davies E, Frogatt K, Higginson I, Kolliakou A. Interventions for improving palliative care for older people living in nursing care homes. Cochrane Database of Systematic Reviews. 2008;2011(3):CD007132.

20. Nicholson C, Evans C, Combes S. Palliative Care, Frailty, and Older People. In: Macleod R, Van den Block L, editors. Textbook of Palliative Care. Cham: Springer Nature Link; 2019. p. 1191–207.

21. Pal LM, Manning L. Palliative care for frail older people. Clinical Medicine, Journal of the Royal College of Physicians of London. 2014;14(3):292–5.

22. Bayly J, Bone AE, Ellis-Smith C, Tunnard I, Yaqub S, Yi D, et al. Common elements of service delivery models that optimise quality of life and health service use among older people with advanced progressive conditions: a tertiary systematic review. BMJ Open. 2021;11(12):e048417.

23. Piers R, Van Den Noortgate N, Vyt A. Collaboration between professionals as a necessary condition for palliative care. In: Van den Block L, Albers G, Martins Pereira S, Onwuteaka-Philipsen B, Pasman R, Deliens L, editors. Palliative care for older people: a public health perspective. Oxford, UK: Oxford University Press; 2015. p. 226–35.

24. Clegg A, Young J, Iliffe S, Rikkert MO, Rockwood K. Frailty in elderly people. The Lancet. 2013;381(9868):752–62.

25. Rockwood K. A global clinical measure of fitness and frailty in elderly people. Can Med Assoc J. 2005;173(5):489–95.

26. Zamora-Sánchez JJ, Urpí-Fernández AM, Sastre-Rus M, Lumillo-Gutiérrez I, Gea-Caballero V, Jodar-Fernández L, et al. The Tilburg Frailty Indicator: A psychometric systematic review. Ageing Res Rev. 2022;76:101588.

27. Rockwood K, Mitnitski A. Frailty in relation to the accumulation of deficits. J Gerontol A Biol Sci Med Sci. 2007;62(7):722–7.

28. Fried LP, Tangen CM, Walston J, Newman AB, Hirsch C, Gottdiener J, et al. Frailty in Older Adults: Evidence for a Phenotype. J Gerontol A Biol Sci Med Sci. 2001;56(3):M146–57.

29. Cesari M, Gambassi G, Van Kan GA, Vellas B. The frailty phenotype and the frailty index: Different instruments for different purposes. Age Ageing. 2014;43(1):10–2.

30. Skoumal M, Honegger M, Roller-Wirnsberger R. Frailty and innovative participatory rehabilitation. Journal of Nutrition, Health and Aging. 2024;28(3):100012.

31. Coventry PA, Grande GE, Richards DA, Todd CJ. Prediction of appropriate timing of palliative care for older adults with non-malignant life-threatening disease: A systematic review. Age Ageing. 2005;34(3):218–27.

32. Kawashima A, Evans CJ. Needs-based triggers for timely referral to palliative care for older adults severely affected by noncancer conditions: a systematic review and narrative synthesis. BMC Palliat Care. 2023;22(1):20.

33. Nicholson CJ, Combes S, Mold F, King H, Green R. Addressing inequity in palliative care provision for older people living with multimorbidity. Perspectives of community-dwelling older people on their palliative care needs: A scoping review. Palliat Med. 2023;37(4):475–97.

34. International Association for Hospice & Palliative Care. Palliative care definition [Internet]. 2025 [cited 2025 Feb 25]. Available from: https://iahpc.org/research/consensus-based-definition-of-palliative-care/definition/

35. Dlima SD, Hall A, Aminu AQ, Akpan A, Todd C, Vardy ERLC. Frailty: a global health challenge in need of local action. BMJ Glob Health. 2024 Aug;9(8):e015173.

36. Hoogendijk EO, Afilalo J, Ensrud KE, Kowal P, Onder G, Fried LP. Frailty: implications for clinical practice and public health. The Lancet. 2019;394(10206):1365–75.

37. Beard JR, Officer A, De Carvalho IA, Sadana R, Pot AM, Michel JP, et al. The World report on ageing and health: A policy framework for healthy ageing. The Lancet. 2016;387(10033):2145–54.

38. Manias E, Bucknall T, Hughes C, Jorm C, Woodward-Kron R. Family involvement in managing medications of older patients across transitions of care: A systematic review. BMC Geriatr. 2019;19(1):95.

39. Ris I, Schnepp W, Mahrer Imhof R. An integrative review on family caregivers’ involvement in care of home-dwelling elderly. Health Soc Care Community. 2019;27(3):e95–111.

40. Van Eechoud IJ, Piers RD, Van Camp S, Grypdonck M, Van Den Noortgate NJ, Deveugele M, et al. Perspectives of family members on planning end-of-life care for terminally ill and frail older people. J Pain Symptom Manage. 2014;47(5):876–86.

41. Van den Block L, Albers G, Martins Pereira S, Onwuteaka-Philipsen B, Pasman R, Deliens L. Palliative care for older people: a public health perspective. Oxford: Oxford University Press; 2015.

42. Stow D, Spiers G, Matthews FE, Hanratty B. What is the evidence that people with frailty have needs for palliative care at the end of life? A systematic review and narrative synthesis. Palliat Med. 2019;33(4):399–414.

43. Looman WM, Fabbricotti IN, Blom JW, Jansen APD, Lutomski JE, Metzelthin SF, et al. The frail older person does not exist: development of frailty profiles with latent class analysis. BMC Geriatr. 2018;18(1):84.

44. Vetrano DL, Palmer K, Marengoni A, Marzetti E, Lattanzio F, Roller-Wirnsberger R, et al. Frailty and Multimorbidity: A Systematic Review and Meta-analysis. The Journals of Gerontology: Series A. 2019;74(5):659–66.

45. Borda MG, Landi F, Cederholm T, Venegas-Sanabria LC, Duque G, Wakabayashi H, et al. Assessment and management of frailty in individuals living with dementia: expert recommendations for clinical practice. Lancet Healthy Longev. 2024;6(1):100666.

46. Dent E, Martin FC, Bergman H, Woo J, Romero-Ortuno R, Walston JD. Management of frailty: opportunities, challenges, and future directions. The Lancet. 2019;394(10206):1376–86.

47. Rykkje LLR, Eriksson K, Raholm MB. Spirituality and caring in old age and the significance of religion - a hermeneutical study from Norway. Scand J Caring Sci. 2013;27(2):275–84.

48. Taube E, Jakobsson U, Midlöv P, Kristensson J. Being in a Bubble: The experience of loneliness among frail older people. J Adv Nurs. 2016;72(3):631–40.

49. Sjöberg M, Beck I, Rasmussen BH, Edberg AK. Being disconnected from life: meanings of existential loneliness as narrated by frail older people. Aging Ment Health. 2018;22(10):1357–64.

50. Nicholson C. A new approach to older people’s end of life care: Living and dying well. Open Access Government [Internet]. 2023 [cited 2025 Mar 25];28–9. Available from: https://www.openaccessgovernment.org/article/new-approach-older-peoples-end-of-life-care-living-dying-well/150392/.

51. Bally ELS, Korenhof SA, Ye L, van Grieken A, Tan SS, Mattace-Raso F, et al. Factors associated with health-related quality of life among community-dwelling older adults: the APPCARE study. Sci Rep. 2024;14(1):14351.

52. Papathanasiou I V, Rammogianni A, Papagiannis D, Malli F, Mantzaris DC, Tsaras K, et al. Frailty and Quality of Life Among Community-Dwelling Older Adults. Cureus. 2021;13:e13049.

53. Kirby SE, Coleman PG, Daley D. Spirituality and Well-Being in Frail and Nonfrail Older Adults. Journal of Gerontology: Psychological Sciences. 2004;59B(3):P123-129.

54. Parker SG, Mccue P, Phelps K, Mccleod A, Arora S, Nockels K, et al. What is Comprehensive Geriatric Assessment (CGA)? An umbrella review. Age Ageing. 2018;47(1):149–55.

55. Garrard JW, Cox NJ, Dodds RM, Roberts HC, Sayer AA. Comprehensive geriatric assessment in primary care: a systematic review. Aging Clin Exp Res. 2020;32(2):197–205.

56. Ellis G, Whitehead MA, Robinson D, O’Neill D, Langhorne P. Comprehensive geriatric assessment for older adults admitted to hospital: Meta-analysis of randomised controlled trials. BMJ (Online). 2011;343(7832):1034.

57. Veronese N, Custodero C, Demurtas J, Smith L, Barbagallo M, Maggi S, et al. Comprehensive geriatric assessment in older people: An umbrella review of health outcomes. Age Ageing. 2022;51(5):afac104.

58. Ekdahl AW, Sjöstrand F, Ehrenberg A, Oredsson S, Stavenow L, Wisten A, et al. Frailty and comprehensive geriatric assessment organized as CGA-ward or CGA-consult for older adult patients in the acute care setting: A systematic review and meta-analysis. Eur Geriatr Med. 2015;6(6):523–40.

59. Aslakson R, Dy S, Wilson R, Waldfogel J, Isenberg S, Blaire A, et al. Assessment Tools for Palliative Care: Technical Brief No. 30 (Prepared by Johns Hopkins University under Contract No. 290-2015-00006-I.) AHRQ Publication No. 14-17-EHC007-EF) [Internet]. Rockville, MD; 2017. Available from: http://www.effectivehealthcare.ahrq.gov/index.cfm/search-for-guides-reviews-and-reports/?pageaction=displayproduct&productid=2442

60. Schmidt EB, Blum D, Domeisen Benedetti F, Schlögl M, Strasser F. Tools for guiding interventions to address patient-perceived multidimensional unmet healthcare needs in palliative care: systematic literature review. BMJ Support Palliat Care. 2023;13(e1):e1.

61. World Health Organisation. Decade of healthy ageing, functional ability, intrinsic capacity decade of healthy ageing baseline report summary [Internet]. Geneva; 2021. Available from: http://apps.who.int/bookorders.

62. Dury S, Dierckx E, van der Vorst A, der Elst M, Fret B, Duppen D, et al. Detecting frail, older adults and identifying their strengths: results of a mixed-methods study. BMC Public Health. 2018;18(1):191.

63. Pan E, Bloomfield K, Boyd M. Resilience, not frailty: A qualitative study of the perceptions of older adults towards “frailty.” Int J Older People Nurs. 2019;14(4):e12261.

64. Nicholson C, Meyer J, Flatley M, Holman C. The experience of living at home with frailty in old age: A psychosocial qualitative study. Int J Nurs Stud. 2013;50(9):1172–9.

65. Nicholson C, Meyer J, Flatley M, Holman C, Lowton K. Living on the margin: Understanding the experience of living and dying with frailty in old age. Soc Sci Med. 2012;75(8):1426–32.

66. Bevilacqua R, Soraci L, Stara V, Riccardi GR, Corsonello A, Pelliccioni G, et al. A systematic review of multidomain and lifestyle interventions to support the intrinsic capacity of the older population. Front Med (Lausanne). 2022;9:929261.

67. Evans N, Pasman HRW, Payne SA, Seymour J, Pleschberger S, Deschepper R, et al. Older patients’ attitudes towards and experiences of patient-physician end-of-life communication: A secondary analysis of interviews from British, Dutch and Belgian patients. BMC Palliat Care. 2012;11.

68. Van Leeuwen KM, Van Loon MS, Van Nes FA, Bosmans JE, De Veti HCW, Ket JCF, et al. What does quality of life mean to older adults? A thematic synthesis. PLoS One. 2019;14(3).

69. Berntsen GKR, Dalbakk M, Hurley JS, Bergmo T, Solbakken B, Spansvoll L, et al. Person-centred, integrated and pro-active care for multi-morbid elderly with advanced care needs: A propensity score-matched controlled trial. BMC Health Serv Res. 2019;19(1):682.

70. Etkind SN, Lovell N, Nicholson CJ, Higginson IJ, Murtagh FEM. Finding a ‘new normal’ following acute illness: A qualitative study of influences on frail older people’s care preferences. Palliat Med. 2019;33(3):301–11.

71. Boa S, Duncan EAS, Haraldsdottir E, Wyke S. Goal setting in palliative care: A structured review. Prog Palliat Care. 2014;22(6):326–33.

72. Tiberini R. Case 35 Goal Setting and Interdisciplinary Support and Care Planning. In: Dewhurst F, Edmonds P, Gillon S, Hawkins A, Miller M, Yardley S, editors. Challenging Cases in Palliative Care. online edition. Oxford University Press; 2024. p. 231–6.

73. Covinsky KE, Eng C, Lui LY, Sands LP, Yaffe K. The last 2 years of life: Functional trajectories of frail older people. J Am Geriatr Soc. 2003;51(4):492–8.

74. Lloyd A, Kendall M, Starr JM, Murray SA. Physical, social, psychological and existential trajectories of loss and adaptation towards the end of life for older people living with frailty: a serial interview study. BMC Geriatr. 2016;16(1):176.

75. Hall A, Boulton E, Kunonga P, Spiers G, Beyer F, Bower P, et al. Identifying older adults with frailty approaching end-of-life: A systematic review. Palliat Med. 2021;35(10):1832–43.

76. White N, Kupeli N, Vickerstaff V, Stone P. How accurate is the “Surprise Question” at identifying patients at the end of life? A systematic review and meta-analysis. BMC Med. 2017;15(1).

77. Theunissen M, Magdelijns FJH, Janssen DJA, Naaktgeboren MW, Courtens A, van Kuijk SMJ, et al. The Surprise Question in Older Hospitalized Patients: To Use or Not to Use? J Am Med Dir Assoc. 2022;23(5):894-896.e1.

78. British Geriatrics Society (BGS). End of Life Care in Frailty: identification and prognostication. 2020; Available from: https://www.bgs.org.uk/resources/end-of-life-care-in-frailty-identification-and-prognostication

79. Downar J, Goldman R, Pinto R, Englesakis M, Adhikari NKJ. The “surprise question” for predicting death in seriously ill patients: a systematic review and meta-analysis. CMAJ. 2017;189:484–93.

80. Rietjens JAC, Sudore RL, Connolly M, van Delden JJ, Drickamer MA, Droger M, et al. Definition and recommendations for advance care planning: an international consensus supported by the European Association for Palliative Care. Lancet Oncol. 2017;18(9):e543–51.

81. van der Steen JT, Nakanishi M, Van den Block L, Di Giulio P, Gonella S, in der Schmitten J, et al. Consensus definition of advance care planning in dementia: A 33-country Delphi study. Alzheimer’s and Dementia. 2023;20(2):1309–20.

82. Sudore RL, Lum HD, You JJ, Hanson LC, Meier DE, Pantilat SZ, et al. Defining Advance Care Planning for Adults: A Consensus Definition From a Multidisciplinary Delphi Panel. J Pain Symptom Manage. 2017;53(5):821-832.e1.

83. Kojima G, Iliffe S, Walters K. Frailty index as a predictor of mortality: A systematic review and meta-analysis. Age Ageing. 2018;47(2):193–200.

84. Dunphy EJ, Conlon SC, O’Brien SA, Loughrey E, O’Shea BJ. End-of-life planning with frail patients attending general practice: An exploratory prospective cross-sectional study. British Journal of General Practice. 2016;66(650):e661–6.

85. Thygesen LC, Christensen K, Rørth M, Sørensen HT, Vandenbroucke JP, Westendorp RGJ. Tipping points – do the prognostic values of multimorbidity and functional status vary with age? Clin Epidemiol. 2021;13:853–7.

86. Crist JD, Liu J, Shea KD, Peterson RL, Martin-Plank L, Lacasse CL, et al. “Tipping point” concept analysis in the family caregiving context. Nurs Forum (Auckl). 2019;54(4):582–92.

87. Carmichael T, Hadžikadić M. The fundamentals of complex adaptive systems. In: Carmichael T, Collins A, Hadžikadić M, editors. Understanding Complex Systems. Cham: Springer; 2019. p. 1–16.

88. Van Rickstal R, De Vleminck A, Engelborghs S, Van den Block L. Experiences with and perspectives on advance care planning in young- and late- onset dementia: A focus group study with physicians from various disciplines. Front Aging Neurosci. 2023;15:1130642.

89. Monnet F, Diaz A, Gove D, Dupont C, Pivodic L, Van den Block L. The perspectives of people with dementia and their supporters on advance care planning: A qualitative study with the European Working Group of People with Dementia. Palliat Med. 2024;38(2):251–63.

90. van der Steen JT, de Wit EJ, Visser M, Nakanishi M, Van den Block L, Korfage IJ, et al. How international experts would define advance care planning: a content analysis. Ann Palliat Med. 2024;13(6):1409–19.

91. Park EJ, Jo M, Park M, Kang SJ. Advance care planning for older adults in community-based settings: An umbrella review. Int J Older People Nurs. 2021;16(5):e12397.

92. Morrison RS, Meier DE, Arnold RM. What’s Wrong with Advance Care Planning? JAMA - Journal of the American Medical Association. 2021;326(16):1575–6.

93. Detering KM, Hancock AD, Reade MC, Silvester W. The impact of advance care planning on end of life care in elderly patients: Randomised controlled trial. BMJ (Online). 2010;340(7751):847.

94. Overbeek A, Korfage IJ, Jabbarian LJ, Billekens P, Hammes BJ, Polinder S, et al. Advance Care Planning in Frail Older Adults: A Cluster Randomized Controlled Trial. J Am Geriatr Soc. 2018;66:1089–95.

95. Sudore R, Hickman S, Walling A. Controversies About Advance Care Planning: Comment & Response. JAMA. 2022;327(7):685.

96. Myers J, Steinberg L, Seow H. Controversies About Advance Care Planning: Comment & Response. JAMA. 2022;327(77):684–5.

97. Mitchell S. Controversies About Advance Care Planning: Comment & Response. 2022;327(7):685–6.

98. Willis D, George D. Are advance care plans of any value? BMJ Support Palliat Care. 2021;11(3).

99. Sharp T, Moran E, Kuhn I, Barclay S. Do the elderly have a voice? Advance care planning discussions with frail and older individuals: A systematic literature review and narrative synthesis. British Journal of General Practice. 2013;63(615):657–68.

100. McMahan RD, Hickman SE, Sudore RL. What Clinicians and Researchers Should Know About the Evolving Field of Advance Care Planning: a Narrative Review. J Gen Intern Med. 2024;39(4):652–60.

101. Frechman E, Dietrich MS, Walden RL, Maxwell CA. Exploring the Uptake of Advance Care Planning in Older Adults: An Integrative Review. J Pain Symptom Manage. 2020;60(6):1208-1222.e59.

102. Lum HD, Sudore RL, Bekelman DB. Advance Care Planning in the Elderly. 2015;99:391–403.

103. Sallnow L, Smith R, Ahmedzai SH, Bhadelia A, Chamberlain C, Cong Y, et al. Report of the Lancet Commission on the Value of Death: bringing death back into life. The Lancet. 2022;399(10327):837–84.

104. Antonides MF, van Wijngaarden E. “It’s like crystal gazing”: The Lived Experience of Anticipating End-of-Life Choices in Older Adults and Their Close Ones. Gerontologist. 2024;64(7):gnae061.

105. Combes S, Nicholson CJ, Gillett K, Norton C. Implementing advance care planning with community-dwelling frail elders requires a system-wide approach: An integrative review applying a behaviour change model. Palliat Med. 2019;33(7):743–56.

106. Weathers E, O’Caoimh R, Cornally N, Fitzgerald C, Kearns T, Coffey A, et al. Advance care planning: A systematic review of randomised controlled trials conducted with older adults. Maturitas. 2016;91:101–9.

107. Hopkins SA, Bentley A, Phillips V, Barclay S. Advance care plans and hospitalized frail older adults a systematic review. BMJ Support Palliat Care. 2020;10(2):1–11.

108. Combes S, Gillett K, Norton C, Nicholson CJ. The importance of living well now and relationships : A qualitative study of the barriers and enablers to engaging frail elders with advance care planning. Palliat Med. 2021;35(6):1137–47.

109. Piers RD, van Eechoud IJ, Van Camp S, Grypdonck M, Deveugele M, Verbeke NC, et al. Advance Care Planning in terminally ill and frail older persons. Patient Educ Couns. 2013;90(3):323–9.

110. Collins P, Hopkins S, Milbourn H, Etkind SN. Uncertainty and advance care planning in older adults living with frailty. A collection and commentary on theme of advanced care planning. Age Ageing. 2024;53(9):afae146.

111. Etkind SN, Li J, Louca J, Hopkins SA, Kuhn I, Spathis A, et al. Total uncertainty: a systematic review and thematic synthesis of experiences of uncertainty in older people with advanced multimorbidity, their informal carers and health professionals. Age Ageing. 2022;51(8).

112. Ellis-Smith C, Tunnard I, Dawkins M, Gao W, Higginson IJ, Evans CJ, et al. Managing clinical uncertainty in older people towards the end of life: a systematic review of person-centred tools. BMC Palliat Care. 2021;20:1–41.

113. Anderson RJ, Stone PC, Low JTS, Bloch S. Managing uncertainty and references to time in prognostic conversations with family members at the end of life: A conversation analytic study. Palliat Med. 2020;34(7):896–905.

114. British Geriatrics Society. End of Life Care in Frailty: Rehabilitation. Available from: https://www.bgs.org.uk/resources/end-of-life-care-in-frailty-rehabilitation

115. Sison SDM, Kim DH. Rethinking emergency care for older adults living with frailty. Lancet Healthy Longev. 2024;5(11):100653.

116. Bone AE, Evans CJ, Etkind SN, Sleeman KE, Gomes B, Aldridge M, et al. Factors associated with older people’s emergency department attendance towards the end of life: a systematic review. Eur J Public Health [Internet]. 2019 Feb [cited 2021 Jun 18];29(1):67–74. Available from: https://academic.oup.com/eurpub/article/29/1/67/5210161

117. Trahan LM, Spiers JA, Cummings GG. Decisions to Transfer Nursing Home Residents to Emergency Departments: A Scoping Review of Contributing Factors and Staff Perspectives. J Am Med Dir Assoc. 2016;17(11):994–1005.

118. Cardona-Morrell M, Kim JCH, Brabrand M, Gallego-Luxan B, Hillman K. What is inappropriate hospital use for elderly people near the end of life? A systematic review. Eur J Intern Med. 2017;42:39–50.

119. Steeman L, Uijen M, Plat E, Huibers L, Smits M, Giesen P. Out-of-hours primary care in 26 European countries: An overview of organizational models. Fam Pract. 2020;37(6):744–50.

120. Low C, Namasivayam P, Barnett T. Co-designing Community Out-of-hours Palliative Care Services: A systematic literature search and review. Palliat Med. 2023;37(1):40–60.

121. National Institute for Health and Care Excellence (NICE). End of life care for adults: service delivery NICE guideline [Internet]. 2019. Available from: www.nice.org.uk/guidance/ng142

122. Holmes S. The distress of uncontrolled pain and symptoms for dying people can’t wait for ‘opening hours’. But there is hope. [Internet]. 2024 [cited 2025 Feb 20]. Available from: https://www.mariecurie.org.uk/blog/palliative-care-out-of-hours

123. Johansson T, Pask S, Goodrich J, Budd L, Okamoto I, Kumar R, et al. Time to care: Findings from a nationally representative survey of experiences at the end of life in England and Wales. Research report. London (UK); 2024.

124. Glans M, Kragh Ekstam A, Jakobsson U, Bondesson Å, Midlöv P. Risk factors for hospital readmission in older adults within 30 days of discharge – a comparative retrospective study. BMC Geriatr. 2020;20(1):467.

125. Tjia J, Velten SJ, Parsons C, Valluri S, Briesacher BA. Studies to reduce unnecessary medication use in frail older adults: A systematic review. Drugs Aging. 2013;30(5):285–307.

126. Chua S, Todd A, Reeve E, Smith SM, Fox J, Elsisi Z, et al. Deprescribing interventions in older adults: An overview of systematic reviews. PLoS One. 2024;19(6):e0305215.

127. Sharkiya SH. Quality communication can improve patient-centred health outcomes among older patients: a rapid review. BMC Health Serv Res. 2023;23(1).

128. Gilmore N, Xu H, Kehoe L, Kleckner AS, Moorthi K, Lei L, et al. Evaluating the association of frailty with communication about aging-related concerns between older patients with advanced cancer and their oncologists. Cancer. 2022;128(5):1101–9.

129. van Vliet LM, Lindenberger E, van Weert JCM. Communication with Older, Seriously Ill Patients. Clin Geriatr Med. 2015;31(2):219–30.

130. Palliative Care Aged Care Evidence. Communication at End of Life: Key messages [Internet]. 2021 [cited 2025 Feb 25]. Available from: https://www.palliaged.com.au/Evidence-Centre/Evidence-Summaries/Communication-at-End-of-Life

131. Elwyn G, Durand MA, Song J, Aarts J, Barr PJ, Berger Z, et al. A three-talk model for shared decision making: Multistage consultation process. BMJ (Online). 2017;359:j4891.

132. Brown EL, Poltawski L, Pitchforth E, Richards SH, Campbell JL, Butterworth JE. Shared decision making between older people with multimorbidity and GPs: A qualitative study. British Journal of General Practice. 2022;72(721):E609–18.

133. Pel-Littel RE, Snaterse M, Teppich NM, Buurman BM, van Etten-Jamaludin FS, van Weert JCM, et al. Barriers and facilitators for shared decision making in older patients with multiple chronic conditions: a systematic review. BMC Geriatr. 2021;21(1):112.

134. van de Pol MHJ, Fluit CRMG, Lagro J, Slaats YHP, Olde Rikkert MGM, Lagro-Janssen ALM. Expert and patient consensus on a dynamic model for shared decision-making in frail older patients. Patient Educ Couns. 2016;99(6):1069–77.

135. Bunn F, Goodman C, Russell B, Wilson P, Manthorpe J, Rait G, et al. Supporting shared decision making for older people with multiple health and social care needs: A realist synthesis. BMC Geriatr. 2018;18(1):165.

136. National Health Service (NHS). Assessing capacity: Consent to treatment [Internet]. 2025 [cited 2025 Mar 24]. Available from: https://www.nhs.uk/conditions/consent-to-treatment/capacity/#:~:text=Capacity%20means%20the%20ability%20to,a%20decision%20at%20that%20time.

137. Stuart RB, Thielke S. Ethical and Practical Ways in Which MOELI (Medical Orders for End-of-Life Intervention) Advance the Physician Orders for Life-Sustaining Treatment (POLST) Program. J Am Med Dir Assoc. 2018;19(3):270–2.

138. Alzheimer Europe. Legal capacity and decision making: The ethical implications of lack of legal capacity on the lives of people with dementia. Luxembourg; 2020.

139. Wendrich-van Dael A, Bunn F, Lynch J, Pivodic L, Van den Block L, Goodman C. Advance care planning for people living with dementia: An umbrella review of effectiveness and experiences. Int J Nurs Stud. 2020;107:103576.

140. Piers R, Albers G, Gilissen J, De Lepeleire J, Steyaert J, Van Mechelen W, et al. Advance care planning in dementia: Recommendations for healthcare professionals. BMC Palliat Care. 2018;17(1).

141. Dening KH, Jones L, Sampson EL. Advance care planning for people with dementia: A review. Int Psychogeriatr. 2011;23(10):1535–51.

142. Boyle G. Recognising the agency of people with dementia. Disabil Soc. 2014;29(7):1130–44.

143. Van Der Byl Williams M, Zeilig H. Broadening and deepening the understanding of agency in dementia. Med Humanit. 2023;49(1):38–47.

144. Biesbrouck T, Jennes DAD, Van Den Noortgate N, De Roo ML. Pharmacological treatment of pain, dyspnea, death rattle, fever, nausea, and vomiting in the last days of life in older people: A systematic review. Palliat Med. 2024;38(10):1088–104.

145. Beernaert K, Smets T, Cohen J, Verhofstede R, Costantini M, Eecloo K, et al. Improving comfort around dying in elderly people: a cluster randomised controlled trial. The Lancet. 2017;390(10090):125–34.

146. Biesbrouck T, De Roo M, Jennes D, Van Den Noortgate N. Guideline for pharmacological treatment of symptoms in the last dats of life of nursing home residents: Validated by the Belgian Evidence-based Medicine Centre (CEBAM) [Richtlijn voor de farmacologische behandeling van symptomen in de laatste levensdagen van woonzorgcentrumbewoners: Gevalideerd door CEBAM]. 2024.

147. Dhollander N, Dierickx S, Eecloo K, Van Den Noortgate N, Deliens L, Beernaert K. Effect of the Care Programme for the Last Days of Life (CAREFuL) on satisfaction with care as perceived by family caregivers and geriatric nurses. A qualitative implementation study. Eur Geriatr Med. 2023;14(4):803–10.

148. Department of Health and Social Care (UK). Review of Liverpool Care Pathway for dying patients: Independent report [Internet]. 2013 [cited 2025 Mar 24]. Available from: https://www.gov.uk/government/publications/review-of-liverpool-care-pathway-for-dying-patients

149. Keeley MP. Family communication at the end of life. Behavioral Sciences. 2017;7(45):1–6.

150. mySupport: Family carer decision support study. The comfort care booklet [Internet]. [cited 2025 Mar 6]. Available from: https://mysupportstudy.eu/about/the-comfort-care-booklet/

151. Chambaere K, Rietjens JA, Smets T, Bilsen J, Deschepper R, Pasman H, et al. Age-based disparities in end-of-life decisions in Belgium: a population-based death certificate survey. BMC Public Health. 2012;12:447.

152. Hug K, Penders YWH, Bischoff-Ferrari HA, Bopp M, Bosshard G. Medical end-of-life decisions in the oldest old in Switzerland. Swiss Med Wkly. 2020;150:w20177.

153. Etkind SN, Bone AE, Lovell N, Higginson IJ, Murtagh FEM. Influences on Care Preferences of Older People with Advanced Illness: A Systematic Review and Thematic Synthesis. J Am Geriatr Soc. 2018;66(5):1031–9.

154. Geiger K, Schneider N, Bleidorn J, Klindtworth K, Jünger S, Müller-Mundt G. Caring for frail older people in the last phase of life - The general practitioners’ view. BMC Palliat Care. 2016;15(1):1–10.

155. Davies N, Mathew R, Wilcock J, Manthorpe J, Sampson EL, Lamahewa K, et al. A co-design process developing heuristics for practitioners providing end of life care for people with dementia. BMC Palliat Care. 2016;15(1):68.

156. National Institute for Health and Care Excellence. Care of dying adults in the last days of life NICE guideline [Internet]. 2015. Available from: www.nice.org.uk/guidance/ng31

157. Hanson S, Brabrand M, Lassen AT, Ryg J, Nielsen DS. What Matters at the End of Life: A Qualitative Study of Older Peoples Perspectives in Southern Denmark. Gerontol Geriatr Med. 2019;5:233372141983019.

158. Harwood RH, Enguell H. End-of-life care for frail older people. BMJ Support Palliat Care. 2019;bmjspcare-2019-001953.

159. Sinclair C, Field S, Williams K, Blake M, Bucks R, Auret K, et al. Supporting decision-making: A guide for people living with dementia, family members and carers. Sydney; 2018.

160. Trialoog. Connection in Trialogue [NL: Verbinding in Trialoog] [Internet]. 2024 [cited 2025 Mar 27]. Available from: https://www.trialooginwzc.be/home

161. The Lancet Healthy Longevity. Caring for our invisible older carers. Lancet Healthy Longev. 2024;5:100662.

162. Ewing G, Grande G. Providing comprehensive, person-centred assessment and support for family carers towards the end of life 10 recommendations for achieving organisational change [Internet]. 2018. Available from: www.hospiceuk.org

163. Ewing G, Grande G. Development of a Carer Support Needs Assessment Tool (CSNAT) for end-of-life care practice at home: A qualitative study. Palliat Med. 2013;27(3):244–56.

164. Anker-Hansen C. The third person in the room: the needs of care partners of older people in home care services. A systematic review from a person-centred perspective. J Clin Nurs. 2018;27(7–8):e1309–26.

165. Lopez-Hartmann M, Wens J, Verhoeven V, Remmen R. The effect of caregiver support interventions for informal caregivers of community-dwelling frail elderly: a systematic review. Int J Integr Care. 2012;12:387–98.

166. Grande GE, Austin L, Ewing G, O’Leary N, Roberts C. Assessing the impact of a Carer Support Needs Assessment Tool (CSNAT) intervention in palliative home care: A stepped wedge cluster trial. BMJ Support Palliat Care. 2017;7(3):326–34.

167. Selman L. Covid grief has cracked us open: How clinicians respond could reshape attitudes to bereavement—an essay by Lucy Selman. The BMJ. 2021;374:n1803.

168. Stroebe M, Stroebe W, Schut. H, Boerner K. Grief is not a disease but bereavement merits medical awareness. The Lancet. 2017;389(10067):389–347.

169. Lundorff M, Holmgren H, Zachariae R, Farver-Vestergaard I, O’Connor M. Prevalence of prolonged grief disorder in adult bereavement: A systematic review and meta-analysis. J Affect Disord. 2017;212:138–49.

170. Boven C, Dillen L, Van den Block L, Piers R, Van Den Noortgate N, Van Humbeeck L. In-Hospital Bereavement Services as an Act of Care and a Challenge: An Integrative Review. J Pain Symptom Manage. 2022;63(3):e295–316.

171. Timmons S, Fox S. Palliative care for people with dementia. In: Miyasaki J, Kluger B, editors. Handbook of Clinical Neurology. Elsevier B.V.; 2023. p. 81–105.

172. de Carvalho I, Epping-Jordan J, Pot AM, Kelley E, Toro N, Thiyagarajan JA, et al. Organizing integrated health-care services to meet older people’s needs. Bull World Health Organ. 2017;95(11):756–63.

173. Van den Block L, Deschepper R, Bilsen J, Van Casteren V, Deliens L. Transitions Between Care Settings at the End of Life in Belgium. JAMA. 2007;298(14):1635–9.

174. Nicholson C, Morrow EM, Hicks A, Fitzpatrick J. Supportive care for older people with frailty in hospital: An integrative review. Int J Nurs Stud. 2017;66:60–71.

175. Briggs AM, Valentijn PP, Thiyagarajan JA, Araujo De Carvalho I. Elements of integrated care approaches for older people: A review of reviews. BMJ Open. 2018;8(4):1–13.

176. Bone AE, Gao W, Gomes B, Sleeman KE, Maddocks M, Wright J, et al. Factors Associated with Transition from Community Settings to Hospital as Place of Death for Adults Aged 75 and Older: A Population-Based Mortality Follow-Back Survey. J Am Geriatr Soc. 2016;64(11):2210–7.

177. Bone AE, Evans CJ, Henson LA, Gao W, Higginson IJ. Patterns of emergency department attendance among older people in the last three months of life and factors associated with frequent attendance: A mortality follow-back survey. Age Ageing. 2019;48(5):676–83.

178. World Health Organization. Integrated care models: an overview [Internet]. Copenhagen; 2016. Available from: http://www.euro.who.int/pubrequest

179. Hendry A, Vanhecke E, Carriazo A, López-Samaniego L, Espinoza J, Sezgin D, et al. Integrated care models for managing and preventing frailty: A systematic review for the European Joint Action on Frailty Prevention (ADVANTAGE JA). Translational Medicine. 2019;19(2):5–10.

180. Evans CJ, Bone AE, Yi D, Gao W, Morgan M, Taherzadeh S, et al. Community-based short-term integrated palliative and supportive care reduces symptom distress for older people with chronic noncancer conditions compared with usual care: a randomised controlled single-blind mixed method trial. Int J Nurs Stud. 2021;103978.

181. de Nooijer K, Pivodic L, Van Den Noortgate N, Pype P, Evans C, Van den Block L. Timely short-term specialized palliative care service intervention for older people with frailty and their family carers in primary care: Development and modelling of the frailty+ intervention using theory of change. Palliat Med. 2021;35(10):1961–74.

182. De Nooijer K, Van Den Noortgate N, Pype P, Pivodic L, Van Den Block L. Timely short-term specialised palliative home care for older people with frailty and their family: A mixed-methods pilot randomised controlled trial and process evaluation. BMJ Open. 2025;15(2).

183. Hoedemakers M, Leijten FRM, Looman W, Czypionka T, Kraus M, Donkers H, et al. Integrated Care for Frail Elderly: A Qualitative Study of a Promising Approach in The Netherlands. Int J Integr Care. 2019;19(3):16.

184. Bone AE, Morgan M, Maddocks M, Sleeman KE, Wright J, Taherzadeh S, et al. Developing a model of short-term integrated palliative and supportive care for frail older people in community settings: perspectives of older people, carers and other key stakeholders. Age Ageing. 2016;45(6):863–73.

185. Hopman P, de Bruin SR, Forjaz MJ, Rodriguez-Blazquez C, Tonnara G, Lemmens LC, et al. Effectiveness of comprehensive care programs for patients with multiple chronic conditions or frailty: A systematic literature review. Health Policy (New York). 2016;120(7):818–32.

186. Frost R, Rait G, Wheatley A, Wilcock J, Robinson L, Harrison Dening K, et al. What works in managing complex conditions in older people in primary and community care? A state-of-the-art review. Health Soc Care Community. 2020;28(6):1915–27.

187. Combes S, Harwood RH, Bramley L, Brookes N, Gordon AL, Laverty D, et al. Building research capacity and capability to enhance the quality of living and dying addressing advancing frailty through integrated care: the ALLIANCE partnership. Public Health Research (Southampt). 2024;30:1–31.

188. Kaiser Permanente Institute for Health Policy. An overview of our integrated care model: Integrated care stories [Internet]. 2025 [cited 2025 Mar 27]. Available from: https://www.kpihp.org/integrated-care-stories/overview/

189. Sadler E, Khadjesari Z, Ziemann A, Sheehan KJ, Whitney J, Wilson D, et al. Case management for integrated care of older people with frailty in community settings. Cochrane Database of Systematic Reviews. 2023;5(5):CD013088.

190. Lupari M, Coates V, Adamson G, Crealey GE. ’We’re just not getting it right’- how should we provide care to the older person with multi-morbid chronic conditions? J Clin Nurs. 2011;20(9–10):1225–35.

191. Metzelthin SF, Van Rossum E, De Witte LP, Ambergen AW, Hobma SO, Sipers W, et al. Effectiveness of interdisciplinary primary care approach to reduce disability in community dwelling frail older people: Cluster randomised controlled trial. BMJ (Online). 2013;347(7926).

192. Goodman C, Dening T, Gordon AL, Davies SL, Meyer J, Martin FC, et al. Effective health care for older people living and dying in care homes: A realist review. BMC Health Serv Res. 2016;16(1):1–14.

193. Rosa WE, Connor S, Ferrell BR, Fulmer T, Travers JL, Radbruch L. Palliation for the frail older adult and what meaningful longevity could be. Lancet Healthy Longev. 2023;4(10):e528–30.

194. Pesut B, Duggleby W, Warner G, Kervin E, Bruce P, Antifeau E, et al. Implementing volunteer-navigation for older persons with advanced chronic illness (Nav-CARE): A knowledge to action study. BMC Palliat Care. 2020 May 22;19(72):1–16.

195. Pesut B, Duggleby W, Warner G, Fassbender K, Antifeau E, Gerard L, et al. Feasibility of a Model of Volunteer Navigation (NCARE) to Support Older Adults Living at Home with Advanced Chronic Illness. J Pain Symptom Manage. 2016;52(6):e36.

196. Pesut B, Duggleby W, Warner G, Bruce P, Ghosh S, Holroyd-Leduc J, et al. A mixed-method evaluation of a volunteer navigation intervention for older persons living with chronic illness (Nav-CARE): findings from a knowledge translation study. BMC Palliat Care. 2020;19(159):1–16.

197. Miranda R, Smets T, Pivodic L, Chambaere K, Pesut B, Duggleby W, et al. Adapting, implementing and evaluating a navigation intervention for older people with cancer and their family caregivers in six countries in Europe: the Horizon Europe-funded EU NAVIGATE project. Palliat Care Soc Pract. 2024;18. 26323524241288873

198. Budde H, Williams GA, Scarpetti G, Kroezen M, Maier CB. What are patient navigators and how can they improve integration of care? HEALTH SYSTEMS AND POLICY ANALYSIS. 2022; Available from: www.euro.who.int

199. Fuller M, Kamans E, van Vuuren M, Wolfensberger M, de Jong MDT. Conceptualizing Empathy Competence: A Professional Communication Perspective. J Bus Tech Commun. 2021 Jul 1;35(3):333–68.

200. Pautex S, Roller R, Katrin W, Nele S, Van Den Noortgate N. Palliative care competencies for geriatricians across Europe: a Delphi consensus study. Eur Geriatr Med. 2021;12(4):817–24.

201. O’Donnell B, Gupta V. Continuous Quality Improvement [Internet]. Treasure Island (FL): StatPearls Publishing; 2023 [cited 2025 Mar 25]. Available from: https://www.ncbi.nlm.nih.gov/books/NBK559239/

202. Patel PM, Deshpande VA. Application Of Plan-Do-Check-Act Cycle For Quality And Productivity Improvement-A Review. Int J Res Appl Sci Eng Technol [Internet]. 2017;5:197–201. Available from: www.ijraset.com

203. Verbeek H, Zwakhalen SMG, Schols JMGA, Kempen GIJM, Hamers JPH. The Living Lab in Ageing and Long-Term Care: A Sustainable Model for Translational Research Improving Quality of Life, Quality of Care and Quality of Work. Journal of Nutrition, Health and Aging. 2020;24(1):43–7.

204. Erasmus School of Health Policy & Management. Reflexive Regulation Using Narrative Approaches:One’s own story as a basis for supervision [Internet]. [cited 2025 Feb 25]. Available from: https://www.eur.nl/en/eshpm/research/research-groups/health-care-governance/projects/regulating-care/ones-own-story-basis-supervision

205. Pot AM, Kok J, Schoonmade LJ, Bal RA. Regulation of long-term care for older persons: a scoping review of empirical research. Int Psychogeriatr. 2024;36(4):289–305.

206. Dubois H, Leončikas T, Molinuevo D, Wilkens M, Llave OV, Weber T, et al. Long-term care workforce: Employment and working conditions [Internet]. Luxembourg; 2020. Available from: www.eurofound.europa.eu

207. Organization for Economic Cooperation and Development. Beyond Applause? Improving Working Conditions in Long-Term Care [Internet]. OECD; 2023. Available from: https://www.oecd.org/en/publications/beyond-applause-improving-working-conditions-in-long-term-care_27d33ab3-en.html

208. Claeys A, Berdai-Chaouni S, Tricas-Sauras S, De Donder L. Culturally Sensitive Care: Definitions, Perceptions, and Practices of Health Care Professionals. Journal of Transcultural Nursing. 2021;32(5):484–92.

209. Gysels M. Cultural issues in palliative care for older people. In: Van den Block L, Albers G, Martins Pereira S, Onwuteaka-Philipsen B, Pasman R, Deliens L, editors. Palliative care for older people: a public health perspective. Oxford: Oxford Academic; 2015.

210. Gysels M, Evans N, Meñaca A, Andrew E, Toscani F, Finetti S, et al. Culture and end of life care: a scoping exercise in seven European countries. PLoS One. 2012;7(4):e34188.

211. Cohen A. The Challenges of Intersectionality in the Lives of Older Adults Living in Rural Areas with Limited Financial Resources. Gerontol Geriatr Med. 2021;7:1–9.

212. Holman D, Walker A. Understanding unequal ageing: towards a synthesis of intersectionality and life course analyses. Eur J Ageing. 2021;18(2):239–55.

213. Hussain JA, Koffman J, Bajwah S. Racism and palliative care. Palliat Med. 2021;35(5):810–3.

214. United Nations. Universal Declaration of Human Rights [Internet]. 1948 [cited 2025 Feb 25]. Available from: https://www.un.org/en/about-us/universal-declaration-of-human-rights

215. United Nations. United Nations Principles for Older Persons [Internet]. 1991 [cited 2025 Feb 25]. Available from: https://www.ohchr.org/en/instruments-mechanisms/instruments/united-nations-principles-older-persons

216. Rosa WE, Ferrell BR, Mason DJ. Integration of Palliative Care into All Serious Illness Care as A Human Right. JAMA Health Forum. 2021;2(4):E211099.

217. Pivodic L, Smets T, Gott M, Sleeman KE, Arrue B, Cardenas Turanzas M, et al. Inclusion of palliative care in health care policy for older people: A directed documentary analysis in 13 of the most rapidly ageing countries worldwide. Palliat Med. 2021;35(2):369–88.

218. Cohen J, Deliens L. A Public Health Perspective on End of Life Care. Online edition. Oxford: Oxford Academic; 2012.

219. De Donder L, Stegen H, Hoens S. Caring neighbourhoods in Belgium: lessons learned on the development, implementation and evaluation of 35 caring neighbourhood projects. Palliat Care Soc Pract. 2024;18:1–14.

220. Pot AM, Rabheru K, Chew M. Person-centred long-term care for older persons: a new Lancet Commission. Vol. 401, The Lancet. Elsevier B.V.; 2023. p. 1754–5.

221. Brassolotto J, Banerjee A. Age-Friendly Communities: Are they also “Friendly” for Death, Dying, Grief, and Bereavement? Canadian Journal on Aging. 2024 Jun 1;43(2):311–8.

222. D’Eer L, Quintiens B, Van den Block L, Dury S, Deliens L, Chambaere K, et al. Civic engagement in serious illness, death, and loss: A systematic mixed-methods review. Palliat Med. 2022;36(4):625–51.

223. Quintiens B, D’Eer L, Deliens L, Van den Block L, Chambaere K, De Donder L, et al. Area-Based Compassionate Communities: A systematic integrative review of existing initiatives worldwide. Palliat Med. 2022;36(3):422–42.

224. Candy B, France R, Low J, Sampson L. Does involving volunteers in the provision of palliative care make a difference to patient and family wellbeing? A systematic review of quantitative and qualitative evidence. Int J Nurs Stud. 2015;52(3):756–68.

225. Woitha K, Hasselaar J, Van Beek K, Radbruch L, Jaspers B, Engels Y, et al. Volunteers in Palliative Care - A Comparison of Seven European Countries: A Descriptive Study. Pain Practice. 2015;15(6):572–9.

226. Horey D, Street AF, O’Connor M, Peters L, Lee SF. Training and supportive programs for palliative care volunteers in community settings. Cochrane Database of Systematic Reviews. 2015;2015(7):CD009500.

227. Dame Cicely Saunders. Volunteers in Hospice and Palliative Care: Handbook for Volunteer Service Managers. Doyle D, editor. Oxford, New York: Oxford University Press; 2002.

228. Burbeck R, Candy B, Low J, Rees R. Understanding the role of the volunteer in specialist palliative care: a systematic review and thematic synthesis of qualitative studies. BMC Palliat Care. 2014;13(1):3.

229. Candy B, Low J, Scott R, Pelttari L. Volunteers in Palliative Care. In: MacLeod R, Van den Block L, editors. Textbook of Palliative Care. Cham: Springer International Publishing; 2018. p. 1–25.

230. Vanderstichelen S, Cohen J, Van Wesemael Y, Deliens L, Chambaere K. The liminal space palliative care volunteers occupy and their roles within it: A qualitative study. BMJ Support Palliat Care. 2020;10(3).

231. Saunders R, Ocampo FA, Graham R, Christiansen A, Gay M, Seaman K. Hospital volunteer programs for older people: A systematic scoping review. Geriatr Nurs (Minneap). 2025;61:185–91.

232. Saunders R, Seaman K, Graham R, Christiansen A. The effect of volunteers’ care and support on the health outcomes of older adults in acute care: A systematic scoping review. J Clin Nurs. 2019;28(23–24):4236–49.

233. Moore A, Motagh S, Sadeghirad B, Begum H, Riva JJ, Gaber J, et al. Volunteer impact on health-related outcomes for seniors: A systematic review and meta-analysis. Canadian Geriatrics Journal. 2021;24(1):44–72.

234. Pesut B, Duggleby W, Warner G, Fassbender K, Antifeau E, Hooper B, et al. Volunteer navigation partnerships: Piloting a compassionate community approach to early palliative care. BMC Palliat Care. 2018;17(2):1–11.

235. Connell B, Warner G, Weeks LE. The Feasibility of Creating Partnerships Between Palliative Care Volunteers and Healthcare Providers to Support Rural Frail Older Adults and Their Families: An Integrative Review. American Journal of Hospice and Palliative Medicine. 2017;34(8):786–94.

236. World Health Organisation. Ageing and health [Internet]. 2024 [cited 2025 Feb 20]. Available from: https://www.who.int/news-room/fact-sheets/detail/ageing-and-health

237. Walsh K, Scharf T, Van Regenmortel S, Wanka A, editors. Social Exclusion in Later Life: Interdisciplinary and Policy Perspectives [Internet]. Vol. 28. Springer Nature; 2021. Available from: http://www.springer.com/series/8818

238. Dahlberg L, McKee KJ, Fritzell J, Heap J, Lennartsson C. Trends and gender associations in social exclusion in older adults in Sweden over two decades. Arch Gerontol Geriatr. 2020;89:104032.

239. Prattley J, Buffel T, Marshall A, Nazroo J. Area effects on the level and development of social exclusion in later life. Soc Sci Med. 2020;246(1):112722.

240. Gendron T, Camp A, Amateau G, Mullen M, Jacobs K, Inker J, et al. The Next Critical Turn for Ageism Research: The Intersections of Ageism and Ableism. Gerontologist. 2024;64(2):1–7.

241. Rabheru K. The Spectrum of Ageism, Mentalism, and Ableism: Expressions of a Triple Jeopardy. American Journal of Geriatric Psychiatry. 2021;29(10):989–92.

242. World Health Organization. Ageing: Ageism. 2021 [cited 2025 Feb 20]; Available from: https://www.who.int/news-room/questions-and-answers/item/ageing-ageismhttps://www.who.int/news-room/questions-and-answers/item/ageing-ageism

243. World Health Organization. Global Report on Ageism. 2021.

244. Greer SL, Lynch JF, Reeves A, Raj M, Gingrich J, Falkenbach M, et al. The politics of healthy ageing: myths and realities [Internet]. Copenhagen; 2022. Available from: http://www.euro.who.int/pubrequest

245. Cluley V, Martin G, Radnor Z, Banerjee J. Talking about frailty: The role of stigma and precarity in older peoples’ constructions of frailty. J Aging Stud. 2021;58:100951.

246. Manuel K, Crotty M, Kurrle SE, Cameron ID, Lane R, Lockwood K, et al. Hospital-Based Health Professionals’ Perceptions of Frailty in Older People. Gerontologist. 2024;64(7):gnae041.

247. Cylus J, Figueras J, Normand C. Will population ageing spell the end of the welfare state: The economics of healthy and active ageing series (EU2019.FI) [Internet]. Copenhagen; 2019. Available from: www.euro.who.int

248. Chao YS, Boivin A, Marcoux I, Garnon G, Mays N, Lehoux P, et al. International changes in end-of-life practices over time: a systematic review. BMC Health Serv Res. 2016;16(1):1–26.

249. Boivin A, Marcoux I, Garnon G, Lehoux P, Mays N, Prémont MC, et al. Comparing end-of-life practices in different policy contexts: a scoping review. J Health Serv Res Policy. 2015;20(2):115–23.

250. Lal A, Erondu NA, Heymann DL, Gitahi G, Yates R. Fragmented health systems in COVID-19: rectifying the misalignment between global health security and universal health coverage. The Lancet. 2021;397(10268):61–7.

251. Ayalon L, Chasteen A, Diehl M, Levy BR, Neupert SD, Rothermund K, et al. Aging in Times of the COVID-19 Pandemic: Avoiding Ageism and Fostering Intergenerational Solidarity. Journals of Gerontology Psychological Sciences and Social Sciences. 2021;76(2):e49-E52.

252. Jiménez-Etxebarria E, Jaureguizar Alboniga-Mayor J, Bernaras Iturrioz E. Cultural Image of Older People during the COVID-19 Pandemic. Int J Environ Res Public Health. 2022;19(22):14644.

253. Braude P, Lewis EG, Broach KC S, Carlton E, Rudd S, Palmer J, et al. Frailism: a scoping review exploring discrimination against people living with frailty. Lancet Healthy Longev. 2025;6(1):100651.

254. Wilkinson DJC. Frailty Triage: Is Rationing Intensive Medical Treatment on the Grounds of Frailty Ethical? American Journal of Bioethics. 2021;21(11):48–63.

255. Lewis EG, Breckons M, Lee RP, Dotchin C, Walker R. Rationing care by frailty during the COVID-19 pandemic. Age Ageing. 2021;50(1):3–6.

256. Cheyne S, Lindley RI, Smallwood N, Tendal B, Chapman M, Navarro DF, et al. Care of older people and people requiring palliative care with COVID- 19: guidance from the Australian National COVID- 19 Clinical Evidence Taskforce. Med J Aust. 2021;216(4):203–8.
